# Electrochemical Deposition of a Single‐Crystalline Nanorod Polycyclic Aromatic Hydrocarbon Film with Efficient Charge and Exciton Transport

**DOI:** 10.1002/anie.202115389

**Published:** 2022-02-03

**Authors:** Cheng Zeng, Wenhao Zheng, Hong Xu, Silvio Osella, Wei Ma, Hai I. Wang, Zijie Qiu, Ken‐ichi Otake, Wencai Ren, Huiming Cheng, Klaus Müllen, Mischa Bonn, Cheng Gu, Yuguang Ma

**Affiliations:** ^1^ State Key Laboratory of Luminescent Materials and Devices Institute of Polymer Optoelectronic Materials and Devices South China University of Technology Guangzhou 510640 P. R. China; ^2^ Max Planck Institute for Polymer Research Ackermannweg 10 55122 Mainz Germany; ^3^ Institute of Nuclear and New Energy Technology Tsinghua University Beijing 100084 P. R. China; ^4^ Shenyang National Laboratory for Materials Science Institute of Metal Research Chinese Academy of Sciences Shenyang 110016 P. R. China; ^5^ Chemical and Biological Systems Simulation Lab Center of New Technologies University of Warsaw Banacha 2C 02-097 Warsaw Poland; ^6^ Institute for Integrated Cell-Material Sciences Institute for Advanced Study Kyoto University Kyoto 606-8501 Japan; ^7^ Guangdong Provincial Key Laboratory of Luminescence from Molecular Aggregates South China University of Technology Guangzhou 510640 P. R. China

**Keywords:** carrier and exciton migration, electrochemical deposition, organic single crystal, orientation control, thin films

## Abstract

Electrochemical deposition has emerged as an efficient technique for preparing conjugated polymer films on electrodes. However, this method encounters difficulties in synthesizing crystalline products and controlling their orientation on electrodes. Here we report electrochemical film deposition of a large polycyclic aromatic hydrocarbon. The film is composed of single‐crystalline nanorods, in which the molecules adopt a cofacial stacking arrangement along the π–π direction. Film thickness and crystal size can be controlled by electrochemical conditions such as scan rate and electrolyte species, while the choice of anode material determines crystal orientation. The film supports exceptionally efficient migration of both free carriers and excitons: the free carrier mobility reaches over 30 cm^2^ V^−1^ s^−1^, whereas the excitons are delocalized with a low binding energy of 118.5 meV and a remarkable exciton diffusion length of 45 nm.

## Graphical abstract

Electrochemical film deposition of a single‐crystalline polycyclic aromatic hydrocarbon on electrodes, with synthetically controlled crystal structure, film thickness, and crystal orientations is reported. The film supports exceptionally efficient migration of both charges and excitons, with a free carrier mobility over 30 cm^2^ V^−1^ s^−1^, a low exciton binding energy of 118.5 meV, and a remarkable exciton diffusion length of 45 nm.

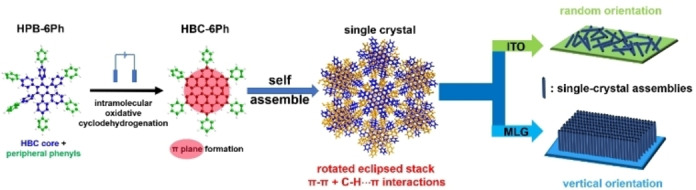

## Introduction

Electroorganic chemistry is a versatile platform for processes such as organic synthesis, battery fabrication, and wastewater treatments.[^1^, ^2^, ^3^] Thin‐film deposition, which is considered a key domain of electroorganic chemistry,^[4]^ has been proven to be a useful technology for preparing electroactive organic semiconductor layers directly on electrodes without involving auxiliary deposition processes. It is thus widely employed in optoelectronic devices based, for example on electrical conductivity, electrochromism, or light emission.[^5^, ^6^, ^7^] Classical conjugated polymers, such as polythiophene, polypyrrole, and polyaniline, can be synthesized and directly deposited as semiconductive thin films on electrodes.[^8^, ^9^, ^10^] While thickness, morphology, and doping state can be controlled, it is difficult to regulate the long‐range order of the molecular packing. Taking the electrochemical synthesis of polythiophene from thiophene as an example,^[11]^ the growing chain length decreases the solubility and thus causes polymer deposition on electrodes.^[12]^ Attempts at aligning polythiophene chains, ultimately under the formation of single crystals,^[13]^ have met with limited success. Electrochemical scanning tunneling microscopy^[14]^ and dip‐pen lithography^[15]^ have been employed toward this end, but only ultra‐thin films (generally a few nm thick) and small domain sizes (hundreds of nm) could be obtained. Another approach, the pretreatment of substrates,[^16^, ^17^] appeared as experimentally demanding, and up to now, only polythiophene has been successfully deposited into single‐crystalline films. Methods of electrochemical deposition under the formation of crystalline polymer films remain challenging but would constitute a major achievement.

In our previous work,[^18^, ^19^] we have developed an electrochemical cyclodehydrogenation method to synthesize various polycyclic aromatic hydrocarbon (PAH) films from oligophenyl precursors and deposit their films into ordered and tightly packed structures. Unlike the electrochemical deposition of conjugated polymers where chain‐growth decreases their solubility, the deposition of PAH films is a consequence of substantially lowered solubility due to the flattening of the aromatic cores. Film characteristics such as thickness, doping level, and aggregation modes could be controlled by the electrochemical conditions, including potential, scan rate, and electrolyte. While this electrochemical method has allowed the synthesis, deposition, and doping of PAH films in one operation, only polycrystalline PAH layers have so far been achieved.

**Figure 1:**
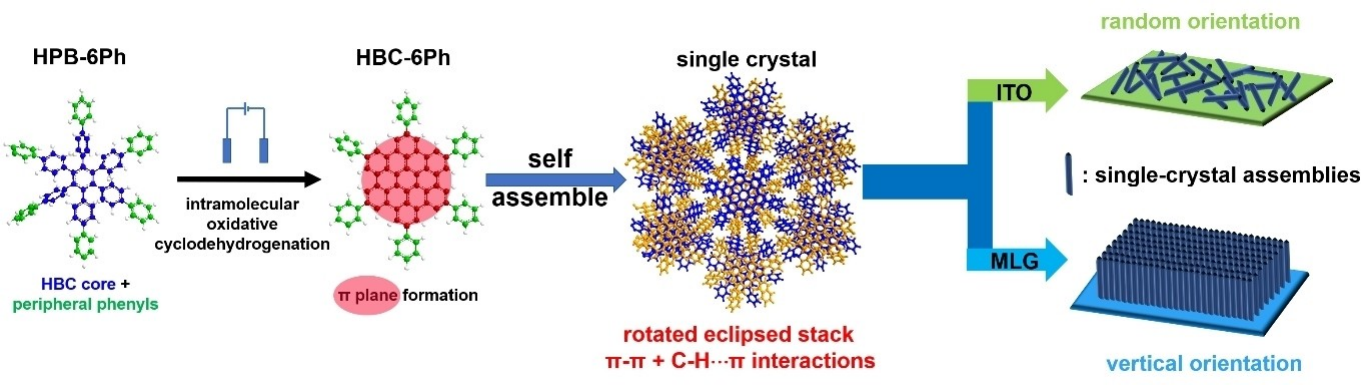
Fabrication of **HBC‐6Ph** single‐crystal films by electrochemical approach. Schematic diagram illustrating the molecular structure of **HPB‐6Ph** and **HBC‐6Ph** and the self‐assemble crystal orientation on ITO or MLG.

Herein, we describe a neutral, single‐crystalline PAH which is deposited on the electrodes as thin films with controllable crystal structure and molecular orientations. The title compound used is 3′′,4′′,5′′,6′′‐tetra([1,1′‐biphenyl]‐4‐yl)‐1,1′ : 4′,1′′ : 2′′,1′′′ : 4′′′,1′′′′‐quinquebenzene (**HPB‐6Ph**), which comprises a central hexaphenylbenzene (**HPB**) moiety and six peripheral phenyl groups. In a three‐electrode electrolytic cell, the oxidative cyclodehydrogenation of the **HPB** units furnishes 2,5,8,11,14,17‐hexaphenylhexabenzo[bc,ef,hi,kl,no,qr]coronene (**HBC‐6Ph**) in single‐crystalline form. Crystal‐structure analysis indicates that the auxiliary intermolecular C−H⋅⋅⋅π interactions among the six outer phenyl groups, synergistically with the π–π interactions, facilitate crystal formation and lock the **HBC‐6Ph** molecules into a single‐crystalline state. Due to the slow diffusion of the **HPB‐6Ph** precursor and the strong interactions among the **HBC‐6Ph** molecules, single‐crystalline nanostructures with lengths up to 500 nm could be deposited on electrodes as homogeneous yet crystalline films (Figure 1). Particularly, by using monolayer graphene (MLG, deposited on conductive substrates) as electrodes, an anisotropic, but uniform face‐on configuration can be achieved with the single‐crystal length of 100 nm. Importantly, both the film morphology and the size of the nanostructures can be synthetically modulated by altering the electrochemical conditions such as scan rate and choice of electrolyte. Such a single‐crystalline PAH film with strong intermolecular interactions leads not only to highly mobile free charge carriers but also to effective dissociation and diffusion of excitons along the crystal lattice.

## Results and Discussion

**Figure 2:**
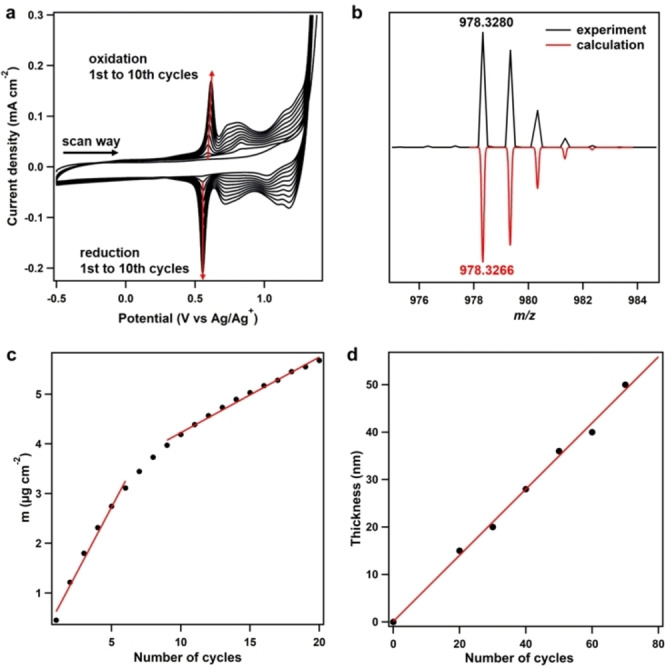
Electrochemical synthesis and deposition of **HPB‐6Ph**. a) CV profiles (1^st^ to 10^th^ cycles) of an **HPB‐6Ph** solution in the presence of the Bu_4_NPF_6_ electrolyte at 25 °C with a scan rate of 0.05 V s^−1^. b) MALDI‐TOF‐MS of **HPB‐6Ph** and **HBC‐6Ph**. c) The plot of the mass density versus the CV cycle number for **HBC‐6Ph**. d) The plot of the film thickness versus the CV cycle number for **HBC‐6Ph**.

The precursor **HPB‐6Ph** (Figure 1) was synthesized via the Co_2_(CO)_8_‐catalyzed cyclotrimerization of 1,2‐di([1,1′‐biphenyl]‐4‐yl)ethyne (Figure S1), and its structure was characterized by NMR and mass spectra (Figures S2, S3). Subsequently, **HPB‐6Ph** was subjected to a three‐electrode electrolytic cell containing dichloromethane as electrolyte solution and 0.1 M Bu_4_NBF_4_, Bu_4_NPF_6_, or Bu_4_NAsF_6_ as supporting electrolytes. The electrochemical synthesis was monitored by cyclic voltammetry (CV) in the potential range of −0.50 to 1.45 V. From the first cycle of the positive scan, the onset potential at 1.28 V accounted for the oxidation of the central **HPB** core with the formation of phenonium (or radical) cations, followed by electrocyclic ring closure under the generation of new aryl–aryl bonds. After that, the resulting positively charged intermediates lost their charge by deprotonation. Consecutive C−C bond‐forming steps could occur until the fully planarized product was formed, which subsequently precipitated from the electrolytes to generate a continuous and homogeneous film due to the decreased solubility. On the subsequent negative scan, reduction peaks at 0.55 and 1.02 V were observed, which could be assigned to the reduction of the positively charged product generated in the former scan to obtain a neutral hydrocarbon (dedoping process). From the second cycle on, new anodic peaks at 0.62, 0.81, and 1.15 V appeared, indicating the oxidation of the newly formed extended PAH at the anode. The multicyclic CV process terminated after the reduction scan in the last cycle and the produced PAH film was neutral. Both anodic and cathodic currents constantly increased with the number of cycles (Figure 2a), illustrating that the product was deposited on the electrode gradually with the cycle number. After 10 cycles, a clear yellow‐green film was generated on an indium tin oxide (ITO) electrode.

The product structure was unequivocally characterized. Matrix‐assisted laser desorption/ionization time‐of‐flight mass spectrometry (MALDI‐TOF MS) (Figure 2b) showed a major peak at *m*/*z*=978.3287, in perfect agreement with the calculated pattern. The central **HPB** core of **HPB‐6Ph**, thus, experienced complete intramolecular cyclodehydrogenation to generate **HBC‐6Ph**, deposited on the anodes. The structural proof was confirmed using Fourier‐transform infrared (FT‐IR) spectroscopy (Figure S4) by the appearance of signals due to C−H twisting vibrations of the 1,3,4,5‐tetrasubstituted phenyl rings (882 cm^−1^) and the disappearance of signals from 1,4‐disubstituted phenyls (858 cm^−1^). Raman spectra showed D (1326 cm^−1^) and G bands (1601 cm^−1^) of **HBC‐6Ph**, which verified the generation of π‐conjugated structures similar to a graphenic core (Figure S5).

To gain more insight into the redox and deposition process, an electrochemical quartz crystal microbalance (EQCM)^[20]^ was employed to detect the mass changes during the electrodeposition. An increase of the mass was observed along with the appearance of the oxidation peaks in the positive scan (doping process) and an obvious decrease in the negative scan (dedoping process). This result indicated that the doping/dedoping process proceeded in parallel with cyclodehydrogenation (Figure S6). Plotting the mass against the scan number revealed a two‐stage increment, with deposition rates of 0.59 and 0.24 μg cm^−2^ for 1–6 and 9–20 cycles, respectively, corresponding to the nucleation and film growth of **HBC‐6Ph** films (Figure 2c). Upon subsequent cycles, the deposition rate remained constant, and each cycle contributed approximately 0.7 nm to the film thickness with a scan rate of 0.1 V s^−1^ (Figure 2d), indicating that the thickness of deposited films can be controlled precisely at a nearly atomic scale. We conducted in situ UV/Vis absorption^[21]^ measurements of the **HBC‐6Ph** films in a precursor‐free electrolyte solution to assess the redox process (Figure S7). The absorption spectra of pristine films showed two major peaks at 373 and 435 nm. On the positive scan from 0 to 1.10 V, the peak at 373 nm was substantially decreased. Together with the disappearance of the absorption peak at 435 nm and a growing broad band from 460 to 600 nm, this result indicated the formation of cationic species and the doping by PF_6_^−^ counterions that were intercalated between **HBC‐6Ph** molecules. Upon the subsequent negative scan, these changes of signal intensities of the absorption peaks were reversed. The shoulder peak at 435 nm reappeared at 0.56 V, suggesting that the molecular stacking structure of **HBC‐6Ph** is recoverable.

**Figure 3:**
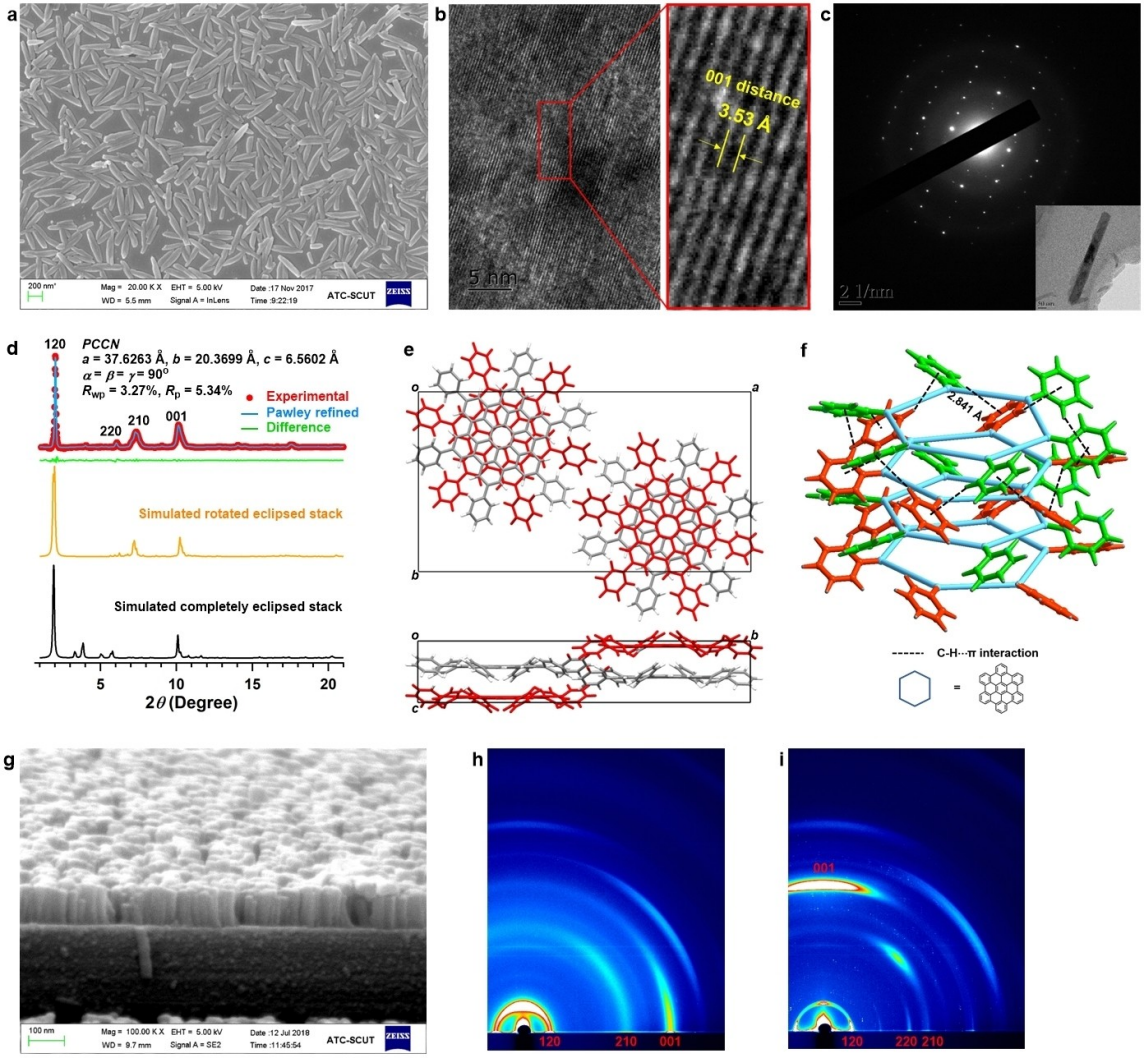
Crystal structure of **HBC‐6Ph** nanorods and orientation control. a) SEM image of **HBC‐6Ph** films on ITO. b) TEM image of the **HBC‐6Ph** nanorods at high magnification. c) SADE pattern of the **HBC‐6Ph** nanorods. d) PXRD pattern: experimentally observed, the simulated rotated eclipsed stacking and the simulated complete eclipsed stacking. e) Top and side views of the **HBC‐6Ph** in the rotated staggered stacking mode. f) C−H⋅⋅⋅π interactions between neighboring peripheral phenyl groups. g) Cross‐section SEM image of **HBC‐6Ph** films on MLG. h) GIWAXS pattern of **HBC‐6Ph** films on ITO. i) GIWAXS pattern of **HBC‐6Ph** films on MLG.

The film morphology was visualized by scanning electron microscopy (SEM) (Figure 3a), which revealed a densely packed crystalline nanorod morphology with a uniform crystal size. Particularly, we found that longer nanorods up to 500 nm could be achieved by applying a slower CV scan rate of 0.05 V s^−1^ or using electrolyte with smaller BF_4_^−^ anion than PF_6_^−^ (Figures S8–S12). On the other hand, parameters including the CV cycles and the concentration of **HPB‐6Ph** also influenced the length of the nanorods (Figures S13, S14). These results illustrated that the electrochemical conditions could regulate the size of the nanorods. High‐resolution transmission electron microscopy (HR‐TEM) measurements of the crystal lattice revealed contiguous lattice fringes arranged along the long axis direction of the nanorods (Figure 3c). The fringe spacing was 3.5 Å, corresponding to the π‐π stacking distance.^[22]^ The selected‐area electron diffraction (SAED) showed a rectangular pattern composed of discrete points and demonstrated the single‐crystal nature of the nanorods (Figure 3b). The interplanar crystal spacing was calculated to be 3.5 Å, which matched well with the TEM results and further proved the compact π–π stacking distance.

The single‐crystal structure of the nanorods was confirmed by synchrotron powder X‐ray diffraction (PXRD) experiments in conjunction with structural simulations. The PXRD pattern of **HBC‐6Ph** exhibited several obvious peaks at 2θ=2.01°, 5.48°, 7.36°, and 10.14°, corresponding to the (120), (220), (210) and (001) facets, respectively (Figure 3d). The (001) facet at 10.14° was related to the π–π distance of 3.5 Å, which matched well with the TEM results. The crystal structure of **HBC‐6Ph** was simulated using density‐functional theory (DFT) (Tables S1 and S2) with the optimized unit‐cell parameters of *a*=37.7 Å, *b*=20.4 Å, *c*=7.0 Å; *α*=*β*=*γ*=90° in a *PCCN* space group, reproducing the experimentally observed pattern with a negligible difference. Notably, the Pawley refinement gave substantially low *R*_wp_ of 3.3 % and *R*_p_ of 5.3 %, demonstrating the reliability of the structural refinements. Based on the unit‐cell parameters, two stacked configurations, namely, completely eclipsed and rotated eclipsed modes, were generated and optimized (Figures 3e, S15). The simulated PXRD pattern of the rotated eclipsed stack model was in good agreement with the experimental pattern, indicating a rotated cofacial stacking. Such a cofacial parallel stacked geometry is rarely observed.^[23]^ Single crystals of most organic small molecules adopt a pitched or rolled π‐stacking mode to minimize the π–π repulsion and avoid steric hindrance.[^24^, ^25^] In our case, the peripheral phenyl substituents between adjacent **HBC‐6Ph** molecules along the π–π direction (with a dihedral angle of 33.31°) generated strong interlayer C−H⋅⋅⋅π interaction with a distance of 2.84 Å, which acted as locks to balance the electrostatic repulsion. This ensured the cofacial stacking **HBC** cores (Figure 3f). The six periphery phenyl units that provide auxiliary intermolecular C−H⋅⋅⋅π interactions are the key to achieving single‐crystalline PAH assemblies. Such cofacial stacking was expected to allow efficient charge transport and high mobility along the π‐interaction direction due to the large π overlap between **HBC‐6Ph** molecular planes.

Besides the combined effect of the strong π–π and C−H⋅⋅⋅π interactions in **HBC‐6Ph**, the diffusion rate of the precursor from the bulk electrolyte solution to the surface of the electrode is crucial for crystal formation. The anode peak currents at 0.64 V and the cathode peak currents at 0.51 V in the CV curves were proportional to the square root of the scan rates ranging from 0.025 to 0.4 V s^−1^ (Figure S16), which demonstrated the redox processes to be determined by the diffusion limit on the electrodes.^[26]^ The diffusion coefficient *D*_0_ of the precursor (from the bulk solution to Helmholtz layer) and the electron‐transfer (losing electrons to anodes) rate *k*_f_ were calculated by Cottrell and Ansonequations, respectively.[^27^, ^28^] We also calculated the same parameters of hexaphenylbenzene as a control (Tables S3). The *k*_f_ of **HPB‐6Ph** was 2.6 cm s^−1^, slightly larger than that of hexaphenylbenzene (2.2 cm s^−1^), profiting from the electron‐donating phenyl groups at the periphery of HBC cores (Figure S17). **HPB‐6Ph** exhibited a low diffusion coefficient *D*_0_ of 1.53×10^−6^ cm^2^ s^−1^, which is 13 times smaller than that of hexaphenylbenzene (2.17×10^−5^ cm^2^ s^−1^) (Figure S18). Therefore, the retarded diffusion of **HPB‐6Ph** can slow down the deposition of **HBC‐6Ph** to a rate favorable for crystal growth.

The orientation of the crystals in a film largely determines the anisotropy of its electronic and optical properties.^[29]^ When using ITO as the anode, **HBC‐6Ph** single crystals grow horizontally along the substrate with a random orientation. This is expected to hamper the directional transport of carriers and excitons. To afford a film in which the **HBC‐6Ph** single crystals were forced into the same orientation, we employed MLG deposited on ITO as anodes because the structural similarity between graphene and PAHs can induce the vertical assembly of PAHs on the MLG surface. The cross‐section SEM image revealed that all the single‐crystalline nanorods exhibited a vertical arrangement on the MLG with uniform sizes of 100 nm (Figure 3g). Notably, only vertically aligned nanorods were observed without any horizontally deposited ones. This suggested an efficient substrate‐induced orientation control that can produce fully oriented nanorod arrays. The different modes of crystal growth of **HBC‐6Ph** nanorods on ITO and MLG were investigated by considering the surface topography of **HBC‐6Ph** deposited on ITO within a few initial CV cycles (Figure S19). It appeared that the nucleation process occurred randomly because of the lattice mismatch between **HBC‐6Ph** single crystals and the ITO electrode, thus causing the subsequent crystal growth to proceed in a random orientation. By contrast, when using MLG as anodes, **HBC‐6Ph** firstly formed spherical and isolated island structures (Figure S20). These islands grew steadily and finally fused to form compact single crystals. Synchrotron grazing‐incidence wide‐angle X‐ray scattering (GIWAXS) of single crystals deposited on ITO displayed an intense π–π stacking peak along the in‐plane direction, indicating an edge‐on arrangement of **HBC‐6Ph** (Figures 3h, S21a). By contrast, the GIWAXS pattern of **HBC‐6Ph** nanorods deposited on MLG exhibited π–π stacking peaks only orthogonal to the surface, demonstrating a single face‐on orientation (Figures 3i, S21b). Therefore, the orientation of **HBC‐6Ph** crystals can be finely regulated by using different anode materials.

**Figure 4:**
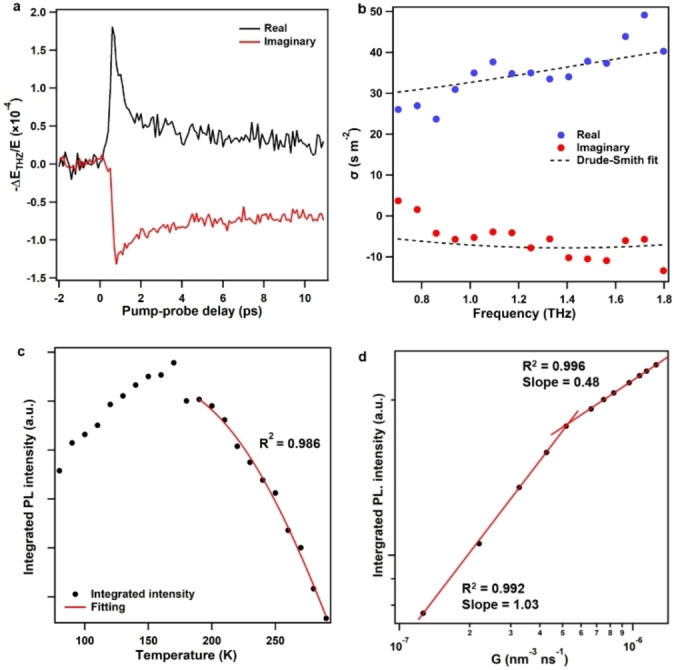
Electronic properties of **HBC‐6Ph** films. a) Time‐resolved photoinduced real and imaginary conductivities of **HBC‐6Ph** films measured as the relative change in terahertz transmission at the peak of the terahertz pulse (real, black line) and the zero‐crossing point of the terahertz pulse (imaginary, red line). b) Frequency‐resolved terahertz conductivity of **HBC‐6Ph** films measured at the peak of the photoconductivity and a Drude–Smith fit. c) Temperature‐dependent integrated PL intensity of **HBC‐6Ph** films on ITO and fits. d) Integrated PL intensity versus exciton generation rate for **HBC‐6Ph** films on ITO.

The unique single‐crystal structure, together with the successful orientation control should, in principle, facilitate long‐range charge transport. Time‐resolved optical pump‐Terahertz (THz) probe spectroscopy^[30]^ was employed to study the photoconductivity of photogenerated charge carriers. For the study, **HBC‐6Ph** single crystals were embedded in a thin, transparent polyacrylic acid (PAA) film. Following the photoexcitation of the sample by a femtosecond laser pulse with a 3.1‐eV photon energy, a fast, sub‐ps rise in both real and imaginary conductivities (Figure 4a) was observed. This ultrafast rise of the conductivities originated from the generation of short‐lived free carriers. To characterize the charge transport, THz time‐domain conductivity measurements at ≈0.6 ps after photoexcitation were conducted. Based on the measurement, we obtained the frequency‐resolved THz complex photoconductivity^[30]^ as shown in Figure 4b, which displays increasing amplitudes for both the (positive) real and (negative) imaginary conductivities with increasing frequency. Such frequency‐dependent photoconductivity is typical for optically‐injected charge carriers in one‐dimensional (1D) carbon‐based nanomaterials, including carbon nanotubes and graphene nanoribbons,[^31^, ^32^] and can be well‐described by a phenomenological model called Drude–Smith (DS) model.^[33]^ In the DS description, the frequency‐dependent conductivity σ(ω) of free charge carriers subject to a preferential backscattering process at e.g. the grain boundary or defects in the materials, is given by Equation 1:

(1) $$\sigma \left(\omega \right)=\frac{\epsilon_{0}\omega_{p}^{2}\tau }{1-i\omega \tau }\left(1+\frac{c}{1-i\omega \tau }\right),\;\mathrm{with}\;\omega_{p}^{2}=\frac{e^{2}N}{\epsilon_{0}m^{{}^{*}}}$$

in which *τ*, *ω_P_*, *ϵ*_0_ and m* are the effective scattering time, the plasma frequency, vacuum permittivity and charge effective mass, respectively. The parameter *c* indicates the probability of backscattering, ranging between 0 (indication of isotropic momentum scattering process) and −1 (representing an anisotropic, full back‐scattering process). Based on the fitting, *c*=−0.71±0.02 was obtained for **HBC‐6Ph** nanorods. This value is fully consistent with previously reported values for films of randomly oriented 1D conductors including carbon nanotubes and graphene nanoribbons.^[31]^ Based on the calculated reduced effective mass m*=0.60 *m*_0_ (Figure S22), the scattering time *τ*=36±7 fs and *c* parameter obtained from the fit to the data in Figure 4b, the charge carrier mobility of the film to be 30.9±6.0 cm^2^ V^−1^ s^−1^ following *μ* (=e*τ*(1+c)/m*). The obtained charge carrier mobility is one of the highest among organic semiconductors,[^34^, ^35^, ^36^, ^37^] which we attribute to the perfect molecular arrangement. In the past, carrier‐transport properties of organic single crystals have been extensively investigated as a function of the prevailing packing modes. For instance, classical 2,6‐diphenylanthracene^[38]^ and rubrene^[39]^ single crystals, with hole mobilities of 34 and 20 cm^2^ V^−1^ s^−1^, respectively, adopt a herringbone packing motif as J aggregates (Table S4). On the other hand, 2,3,9,10‐tetrachloro‐6,13‐bis((triisopropylsilyl)ethynyl)quinoxalino[2,3‐b]phenazine (4Cl‐TAP)^[40]^ and 2,2′‐(2,5‐difluorocyclohexa‐2,5‐diene‐1,4‐diylidene)dimalononitrile (F2‐TCNQ)^[41]^ single crystals show a staggered dense packing with a minimal π–π distance (Table S4), thus achieving a band‐like transport with hole mobilities of 28 and 25 cm^2^ V^−1^ s^−1^, respectively. Organic single crystals with cofacial stacking are much less reported. Among crystals with cofacial arrangements, the hole mobilities of 3,7‐bis((*E*)‐1‐(2‐ethylhexyl)‐5,7‐difluoro‐2‐oxoindolin‐3‐ylidene)‐3,7‐dihydrobenzo[1,2‐b:4,5‐b′]difuran‐2,6‐dione (F_4_‐BDOPV)^[42]^ and (2Z,2′Z)‐2,2′‐(1,4‐phenylene)bis(3‐(thiophen‐3‐yl)acrylonitrile) (α‐PBTA)^[43]^ reached 12.6 and 2.7 cm^2^ V^−1^ s^−1^, respectively (Table S4). Compared with the above crystal packing modes and their benchmark carrier mobilities, our system compares favorably with previously reported cofacial single crystals. We note that the mobility μ quantified by THz measurements represents the short‐range μ as charge carriers are locally (up to 10s of nm) driven by the rapidly oscillating THz field.^[44]^

While the THz measurements on the initial free charges generated by optical excitation unveil high carrier mobilities for free electrons and holes, a large portion of free charges evolves into excitons owing to the low dielectric constant of carbon‐based materials. Excitons do not contribute to real conductivity, but their polarizability contributes to the imaginary conductivity. Indeed, the imaginary conductivity dominates the dynamics at later times (after ≈5 ps, as shown in Figure 4a). To determine the exciton binding energy and diffusion length, we performed temperature‐dependent and power‐dependent photoluminescence measurements of the **HBC‐6Ph** thin films.[^45^, ^46^] The steady‐state photoluminescence spectra of **HBC‐6Ph** films upon excitation at 370 nm showed an apparent broad peak at 545 nm, corresponding to the π*‐π transition process from the S_1_ to the S_0_ state. The photoluminescence intensity gradually increased when lowering the temperature, indicating that the emission probability of the photoinduced charge carriers was thermally activated. This can be understood by the fact that with lowering temperatures (and thus the thermal excitations), more charge carriers condensate into exciton states, leading to photoluminescence enhancement.^[47]^ The exciton binding energies (E_B_) of **HBC‐6Ph** films deposited on ITO and MLG extracted from the fitting of an Arrhenius equation were 118.5±9.4 and 41.7±3.2 meV, respectively (Figures 4c, S23–S25). These values are substantially lower than those of most organic semiconductors with E_B_ generally ranging from 500 to 2000 meV (Tables S5). The packing into a crystal may enhance the charge screening effect between photogenerated carriers, and thus reduce the exciton binding energy. Power‐dependent measurements of an **HBC‐6Ph** thin film revealed that the photoluminescence intensity increased steadily with the excitation power density (Figures S26, S27). A log‐log plot of the integrated PL intensity versus excitation density displayed two slopes of 1.0 and 0.5 with a boundary at 5×10^−7^ nm^−3^ ns^−1^, which revealed additional fluorescence quenching from the exciton‐exciton annihilation (Figures 4d, S28).^[48]^ Based on the threshold of the exciton generation rate for annihilation, the exciton diffusion lengths of **HBC‐6Ph** films deposited on ITO and MLG are calculated to be 45 and 63 nm, respectively. These values were substantially higher than those of most organic semiconductors, whose exciton diffusion lengths generally ranged from 1 to 30 nm (Tables S6). These results indicated unobstructed exciton migration over a large distance along the crystal lattice. Such PAH film enabling efficient migration of both free carriers and excitons should be promising for many optoelectronic device applications. For instance, previous works have already demonstrated good device performances in field‐effect transistors and photovoltaics by using PAH derivatives.[^49^, ^50^, ^51^]

## Conclusion

In summary, we have electrochemically synthesized a single‐crystalline PAH, which could be deposited on electrodes in situ to form homogeneous thin films. The synergy of strong π–π and C−H⋅⋅⋅π interactions and the slow diffusion of precursors were essential in promoting the formation of single crystals. All key characteristics such as film thickness, crystal size, and molecular orientation could be controlled by an appropriate choice of electrochemical conditions. The **HBC‐6Ph** film exhibited high carrier mobility of over 30 cm^2^ V^−1^ s^−1^, low exciton binding energy of 41.7±3.2 meV, and extended exciton diffusion length of 63 nm. The above results indicated superior transport characteristics for both free carriers and excitons in the **HBC‐6Ph** film. Owing to the advantages of facile synthesis and successful control over crystal structures and orientations by electrochemical deposition, we anticipate that this method will provide an important protocol for synthesizing single‐crystalline arrays of organic semiconductors directly on electrodes, by exploring other precursors and improved electrochemical conditions. Such high‐mobility arrays promise improved performances of optoelectronic devices such as photodetection and photocatalysis, where long‐range diffusion length for excitons with a high dissociation efficiency at interfaces and high mobility for free carriers for their transport towards electrodes are crucial.

## Conflict of interest

The authors declare no conflict of interest.

1

## Supporting information

As a service to our authors and readers, this journal provides supporting information supplied by the authors. Such materials are peer reviewed and may be re‐organized for online delivery, but are not copy‐edited or typeset. Technical support issues arising from supporting information (other than missing files) should be addressed to the authors.

Supporting Information

## Data Availability

The data that support the findings of this study are available from the corresponding author upon reasonable request.
