# Supplementary material for: Electrochemical Deposition of a Single‐Crystalline Nanorod Polycyclic Aromatic Hydrocarbon Film with Efficient Charge and Exciton Transport
Source: Angew Chem Int Ed Engl. 2022 Feb 3;61(13):e202115389. doi: 10.1002/anie.202115389 (PMC9306484; doi:10.1002/anie.202115389)
Supplement: Supplementary file 1 — Supporting Information [file ANIE-61-0-s001.pdf]

Angewandte Chemie
Eine Zeitschrift der Gesellschaft Deutscher Chemiker

Supporting Information

Electrochemical Deposition of a Single-Crystalline Nanorod Polycyclic Aromatic Hydrocarbon Film with Efficient Charge and Exciton Transport

C. Zeng, W. Zheng, H. Xu, S. Osella, W. Ma, H. I. Wang, Z. Qiu, K.-i. Otake, W. Ren*, H. Cheng*, K. Müllen*, M. Bonn*, C. Gu*, Y. Ma**

SUPPORTING INFORMATION

Table of Contents

Experimental Procedures……S3
Results and Discussion……S6
References……S49
Author Contributions……S51

Experimental Procedures

Materials and methods

Materials

Dichloromethane (DCM), 1,4-dioxane, n-hexane, methylbenzene, $K_3Fe(CN)_6$, KCl, Bu_4NBr, Bu_4NPF_6 and Bu_4NBF_4 were purchased from J&K chemical. Dicobaltoctacarbonyl ($Co_2(CO)_8$) and $KAsF_6$ were purchased from TCI. Polyacrylic acid solution (PAA) (average Mw ~250,000, 35 wt% in H_2O) was purchased from Sigma Aldrich. 4-(2-[1,1'-Biphenyl]-4-ylethynyl)-1,1'-biphenyl was purchased from Energy Chemical. Bu_4NAsF_6 was synthesized by replacement reaction from Bu_4NBr and $KAsF_6$.

Methods

Synthesis of HPB-6Ph: 4-(2-[1,1'-Biphenyl]-4-ylethynyl)-1,1'-biphenyl (100 mg, 0.30 mmol) and dicobaltoctacarbonyl (0.3 mg, 8.7 × 10^{-4} mmol) were mixed in 1,4-dioxane (10 mL) in an 80-mL high-pressure-resistant tube under the nitrogen atmosphere in glove box. The tube was sealed and heated at 125 °C for 48 h. The mixture was cooled to room temperature and the resulting precipitate was washed with n-hexane for 3 times. Afterwards, the solid was dispersed in dichloromethane, and the supernatant solution was collected by centrifugation. The solution was dried by vacuum evaporation to obtain the crude product, which was subjected to recrystallization with toluene for 24 h to produce **HPB-6Ph** (58 mg) as a white powder in a 58% isolated yield. ^1H NMR (500 MHz, CD_2Cl_2, ppm): δ= 7.42 (d, J = 7.4 Hz, 12H), 7.30 (t, J = 7.6 Hz, 12H), 7.21 (dd, J = 15.8 Hz, 7.8 Hz, 18H), 7.03 (d, J = 8.2 Hz, 12H). MS m/z: calc. 990.42, found 990.44.

Preparation of HBC-6Ph films: The **HBC-6Ph** films were electrochemically synthesised from **HPB-6Ph** (1.01 × 10^{-4} mol L^{-1} in electrolyte solution) in a three-electrode system using glassy carbon, indium tin oxide (ITO) or monolayer graphene (MLG, deposited on ITO) as the working electrode, a Pt wire or titanium plate as the counter electrode, and an Ag/Ag^+ (ACN containing 0.01 M $AgNO_3$ and 0.1 M Bu_4NPF_6 was used as internal reference solution) as the reference electrode. DCM with supporting electrolyte (0.1 M Bu_4NPF_6, Bu_4NPF_6 or Bu_4NAsF_6) was used as electrolyte solution. Electrochemical oxidation was conducted by multicycle CV in the potential range from -0.5 to 1.45 V. In addition to applying the CV method, the **HBC-6Ph** films were also prepared on ITO by potentiostatic method of precursors at 1.45 V, followed by applying a potential at -0.5 V for 200 s to neutralize the cationic species. This method yields micrometer-thick films that can be easily scraped off the electrodes to form powders with a sufficient mass for analysis. After the deposition was completed, the films were washed with DCM to remove doped counterions. All electrochemical experiments were carried out in a glove box (nitrogen atmosphere). The product structures were characterized by matrix-assisted laser-desorption time-of-flight mass spectrometry (MALDI-TOF MS), Fourier transform infrared (FT-IR), and Raman spectroscopy.

General instrumental analysis

^1H NMR spectra were recorded on a Bruker AVANCE HD III 500M NMR spectrometer, where the chemical shifts (δ in ppm) were determined with respect to tetramethylsilane (TMS) as an internal reference. The MALDI measurements for solid-state samples were carried out on a rapifleXTM MALDI-TOF/TOF mass spectrometer from Bruker Daltonik GmbH using 7,7,8,8-tetracyanoquinodimethane (TCNQ) as the matrix. Fourier transform Infrared (FT-IR) spectra were recorded on an IFS 66V/S Fourier transform infrared spectrophotometer. UV-vis spectra were recorded on a Shimadzu UV-3600 spectrometer. Synchrotron powder X-ray diffraction (PXRD) data were recorded on beamline BL15U1, Shanghai Synchrotron Radiation Facility (SSRF), P. R. China, with a wavelength of 0.6199 Å, from 2θ = 2° up to 30° with 0.01° increment. Field-emission scanning electron microscopy (FE-SEM) was performed on a Zeiss Merlin operating at an accelerating voltage of 5.0 kV. High-resolution transmission electron microscopy (HR-TEM) images were obtained on a TEM JEOL 2100F with an acceleration voltage of 300 kV. Photoluminescence spectra were recorded on a Jobin-Yvon Spex Fluorolog-3 spectrofluorometer. The film thickness was recorded on a Veeco Dektak 150 atomic profiler. The synchrotron grazing-incidence wide-angle X-ray scattering (GIWAXS) experiments were conducted at the BL02B2 line at SPring-8, Japan.

Fabrication of monolayer graphene deposited on ITO

Monolayer graphene film was first grown on Pt foil by chemical vapor deposition (CVD) and then transferred onto ITO/quartz substrate by a bubbling transfer method, as reported previously.[S1] To grow graphene film, a piece of Pt foil (250 μm-thick, 99.99 wt% metal basis, 20 mm × 10 mm) was loaded into a fused-silica tube (inner diameter: 22 mm), heated to 1100 ℃ under the protection of H_2, and then annealed at 1100 ℃ for 10 min to remove residual carbon or organic substances. After that, graphene growth was initiated and maintained for 20 min under a mixture of CH_4 (3 sccm) and H_2 (600 sccm). To transfer monolayer graphene film onto ITO/quartz

substrate, the as-grown monolayer graphene film on Pt foil was spin-coated with PMMA (950 kDa molecular weight, 4 wt% in ethyllactate) at 2000 r. p. m. for 1 min, and then cured at 90 °C for 10 min. The PMMA/graphene stack was separated from the Pt foil in a 1 M NaOH aqueous solution under a constant current of 0.2 A, and then collected onto the ITO/quartz substrate and baked at 130 °C for 1 h to remove the residual water. After PMMA was removed by acetone at room temperature, the resulting graphene/ITO/quartz was annealed at 300 °C for 5 h under a pressure of 10^{-5} Pa to further clean the surface of graphene.

Active electrode surface area

The active surface areas (non-apparent area) of glassy carbons were calibrated as follows: the electrodes to be tested were placed in an aqueous solution containing $K_3Fe(CN)_6$ (5.0×10^{-3} mol L^{-1}) and KCl (1 mol L^{-1}), and the CV curves were recorded in the range of -0.25 to 0.75 V (vs. Ag/AgCl). Based on Randles–Sevcik equation:[S2]

$$i_p = 2.69 \times 10^5 A n^{3/2} D_0^{1/2} C_0 v^{1/2}$$

where i_p is reduction peak current, A is active surface area of electrode, n is electron-transfer number, C_0 is bulk concentration of $K_3Fe(CN)_6$, D_0 is diffusion coefficient and v is scan rate. In this case,[S3] $D_0 = 7.6 \times 10^{-6}$ $cm^2\,s^{-1}$ (298 K); $n = 1$; $C_0 = 5.0 \times 10^{-3}$ mol L^{-1}; $v = 0.05$ V s^{-1}.

Cottrell equation

The Cottrell equation,[S4] describes the chronoamperometric response, and its integral from t = 0 gives the cumulative charge passed in reducing the diffusing reactant:

$$Q = \frac{2nFAD_0^{1/2} C_0 t^{1/2}}{\pi^{1/2}}$$

where Q is electricity, n is transfer electron number, F is faraday constant, A is active surface area of electrodes, C_0 is bulk concentration of the electroactive units and t is charging time. The diffusion rate D_0 can be found from the linear slope in the time scale of 10 s.

Anson equation

The Anson equation[S5] is also useful to get a fully or partially controlled chronocoulometric response by interface charge transfer kinetics. This goal can be achieved by using a step potential that performs diffusion control electrolysis less aggressively throughout the experimental time domain. In other words, it is necessary to perform a step on the potential in the rising portion of the sampled current voltammogram, which corresponds to the time scale of interest, and the time scale must be short enough that the electrode dynamics control the current for a relatively long period of time. If a step at t = 0 from an initial potential where electrolysis does not occur, Anson equation can be obtained:

$$Q = nFAk_f C_0 \left(\frac{2t^{1/2}}{H\pi^{1/2}} - \frac{1}{H^2} \right)$$

where Q is electricity, n is transfer electron number, F is faraday constant, A is active surface area of electrodes, C_0 is bulk concentration of the electroactive units and t is charging time. The electron transfer rate K_f can be found from the linear slope in the time scale of 0.1 s.

Density-functional theory simulation

The crystalline structures of **HBC-6Ph** were calculated using density-functional theory (DFT) implemented in the CASTEP module of Materials Studio 7.0.[S6,S7] The generalized gradient approximation (GGA) in the form of Perdew–Burke–Ernzerhof (PBE)[S8,S9] was selected as the exchange-correlation functional. Grimme dispersion correction[S10,S11] was employed in all calculations to describe van der Waals (vdW) and π-stacking interactions. The lattice dimensions were optimized simultaneously with the geometry. A plane wave energy cutoff of 750 eV and the Monkhorst-Pack *k*-point grid of 1 × 1 × 2 were used. The convergence criteria for energy, force, stress and displacement are 5×10^{-6} eV/atom, 0.01 eV/Å, 0.02 GPa and 5×10^{-4} Å, respectively.

Preparation of HBC-6Ph films on PAA for THz measurements

The HBC-6Ph films were firstly deposited on ITO or MLG, and then uniformly coated with an aqueous solution of PAA (average Mw ~250,000, 35 wt% in H_2O). After the water evaporated and dried, the PAA on the surface of HBC-6ph films was peeled off, thus the HBC-6ph films were transferred to the PAA substrates from ITO or MLG.

THz spectroscopy

The optical pump-THz probe spectrometer is operated by a regenerative Ti: sapphire femtosecond amplifier system with ~50 fs duration pulsed laser. The center wavelength of pulses is ~ 800 nm and the repetition rate is 1 kHz. A single-cycle THz pulse in the 0-2.5 THz frequency range is generated by an optical rectification process by pumping a 0.5-mm thick ZnTe crystal by 800 nm pulses. The transient THz electric field can be mapped out via electro-optic sampling by a third pulse (800 nm, 50 fs duration)[S12] by varying the arrival of the sampling pulse to the detection crystal with respect to the THz pulse. In order to monitor the charge carrier dynamics, the sample was photo-excited by 400 nm pulse. The conductivity of photogenerated charge carriers, i.e., photoconductivity can be further measured by tracking the photo-induced THz field absorption.

Calculation of effective mass of HBC-6Ph single crystal

The **HBC-6Ph** crystal structure was considered as input geometry for computation of the effective mass, with the Quantum Espresso 6.5 suite of programs.[S13] Ultrasoft pseudopotentials,[S14] together with PBE[S15] functional and with 25 and 200 Ry cut-offs for wavefunctions and charge density respectively, were used. Since van der Waals interactions play a major role in the stability of the assembly, vdw-DF2 term for the exchange and correlation was used.[S16] Dipole corrections were applied to maintain a constant, zero external electric field and minimize the electrostatic potential fluctuations. To maintain the crystal structure, a single point calculation was performed at the Gamma point of the first Brillouin zone. The band dispersion along the *c* direction of the crystal structure was obtained in a subsequent calculation in which the vicinity of the valence band maximum and conductive band minimum were considered, by reading k-points in crystal coordinates. The computed effective masses are $m_h = 0.69\ m_0$ for holes and $m_e = 4.33\ m_0$ for electrons, leading to a total reduced effective mass of $m^* = 0.60$.

Exciton binding energy

The PL intensity verse temperature plot could be fitted using

$$I(T) = \frac{I_0}{I + Ae^{(-E_B/k_BT)}}$$

in which I_0 is the PL intensity at 0 K, k_B is the Boltzmann constant and E_B is exciton binding energy.

Exciton-exciton annihilation analysis

Exciton-exciton annihilation occurs ($S_1 + S_1 \rightarrow S_n + S_0 \rightarrow S_1 + S_0$ + phonon) under excitation by intermediate or high photo fluxes, where the fusion of two excitations forms ionized or higher-energy state. In the case of singlet-singlet annihilation, the kinetic equation is written by following formula:[S17]

$$\frac{dN(t)}{d(t)} = G - \frac{N(t)}{\tau} - \gamma N^2(t)$$

where *N(t)* is the exciton density at a specific time, *G* is the generation rate of exciton density (excitation density), τ is the intrinsic lifetime of the excitons, and γ is the annihilation rate. Under steady laser excitation, the continuous generation of excitons makes the left term being zero. Thus, the constant solution of above equation is $N = ((1 + 4\gamma\tau^2 G)^{1/2} - 1)/2\gamma\tau$. There are two straight lines with slopes of 1 and 0.5 in a log-log graph. The crossing point is defined as the threshold generation rate (G_0), which is estimated as $G_0 \approx (\gamma\tau^2)^{-1}$. For **HBC-6Ph** films deposited on ITO, G_0 was extracted to be 5.77×10^{-7} nm^{-3} s^{-1}, and thus γ was calculated to be 3.74×10^{-7} cm^3 s^{-1}. The diffusion constant (*D*) was then calculated as 0.298 cm^2 s^{-1} according to the relation $D = \gamma/(4\pi Ra)$, where *Ra* is annihilation radius and is set to be 1 nm. On the basis of the above theoretical analysis that the exciton migration in the bulk phase of **HBC-6Ph** follows a one-dimensional model, the exciton diffusion length (*L*) was calculated to be 45 nm by $L = (\tau D)^{1/2}$. For **HBC-6Ph** films deposited on MLG, G_0 was extracted to be 1.34×10^{-6} nm^{-3} s^{-1}, and thus γ was calculated to be 3.32×10^{-7} cm^3 s^{-1}. The diffusion constant (*D*) was then calculated as 0.264 cm^2 s^{-1} according to the relation $D = \gamma/(4\pi Ra)$, where *Ra* is annihilation radius and is set to be 1 nm. On the basis of the above theoretical analysis that the exciton migration in the bulk phase of **HBC-6Ph** follows a one-dimensional model, the exciton diffusion length (*L*) was calculated to be 63 nm by $L = (\tau D)^{1/2}$.

Results and Discussion

Figures

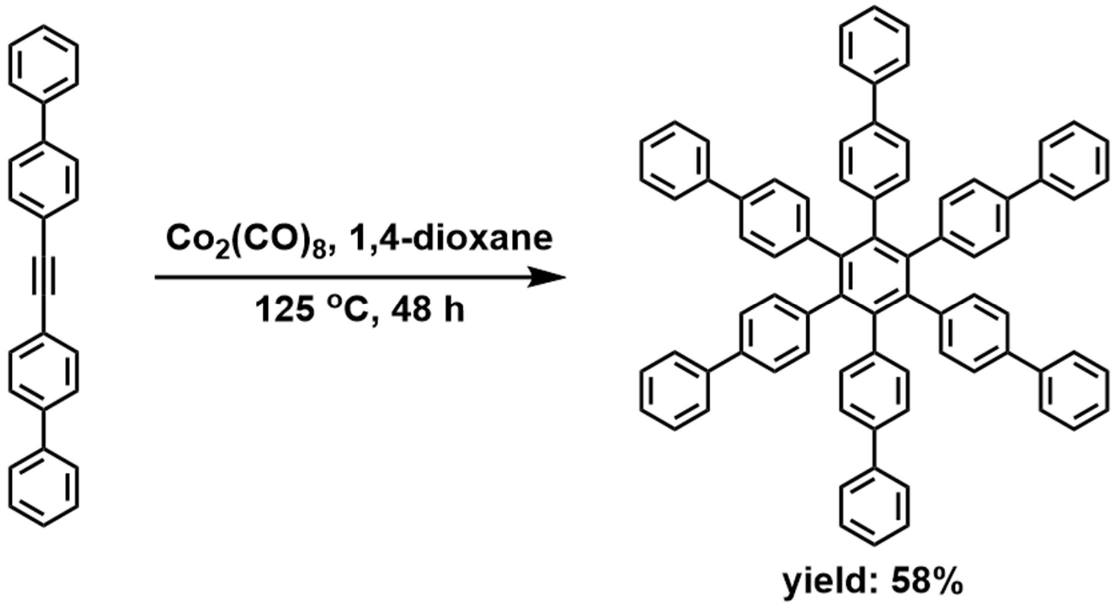

Figure S1. Synthesis of **HPB-6Ph**.

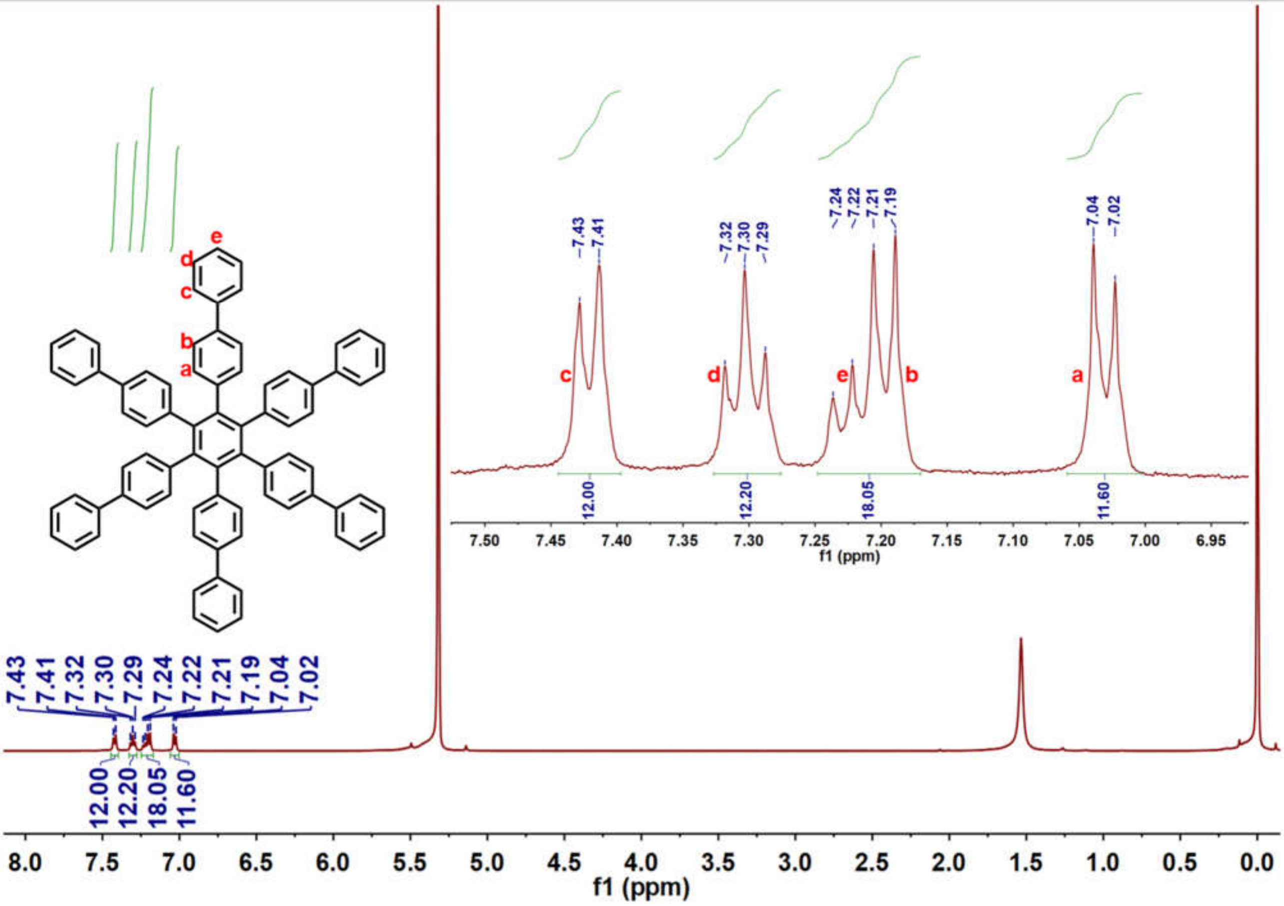

Figure S2. ^{1}H NMR spectra of **HPB-6Ph**.

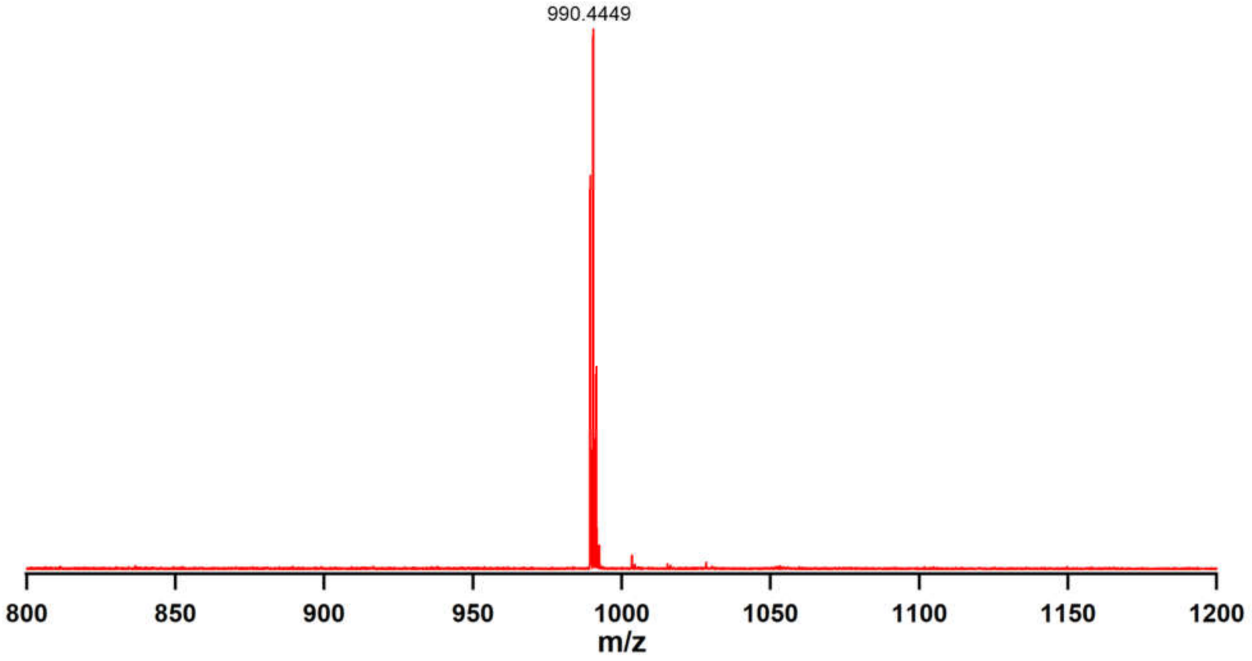

Figure S3. MALDI-TOF mass spectrum of **HPB-6Ph**.

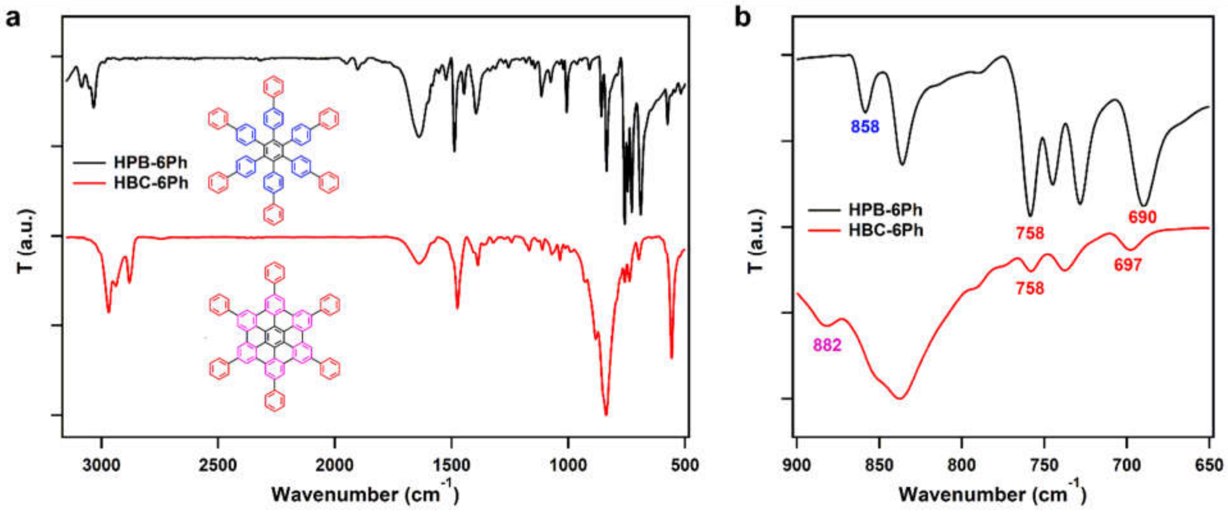

Figure S4. FT-IR spectra of **HPB-6Ph** and **HBC-6Ph** powder. (a) 3250-500 cm^{-1}. (b) 900-650 cm^{-1}. For **HPB-6Ph**, the medium strong bands appeared at 758 and 690 cm^{-1} (red) were contributed to C–H twisting vibrations of the monosubstituted phenyl rings. The peaks located at 858 cm^{-1} (blue) were produced by the C–H twisting vibrations of the 1,4-disubstituted benzenes. For **HBC-6Ph**, the peaks located at 758 and 697 cm^{-1} (red) were produced by the C–H twisting vibrations of the monosubstituted phenyl rings. The peak located at 882 cm^{-1} was produced by the C–H twisting vibrations of the 1,3,4,5-tetrasubstituted benzenes.

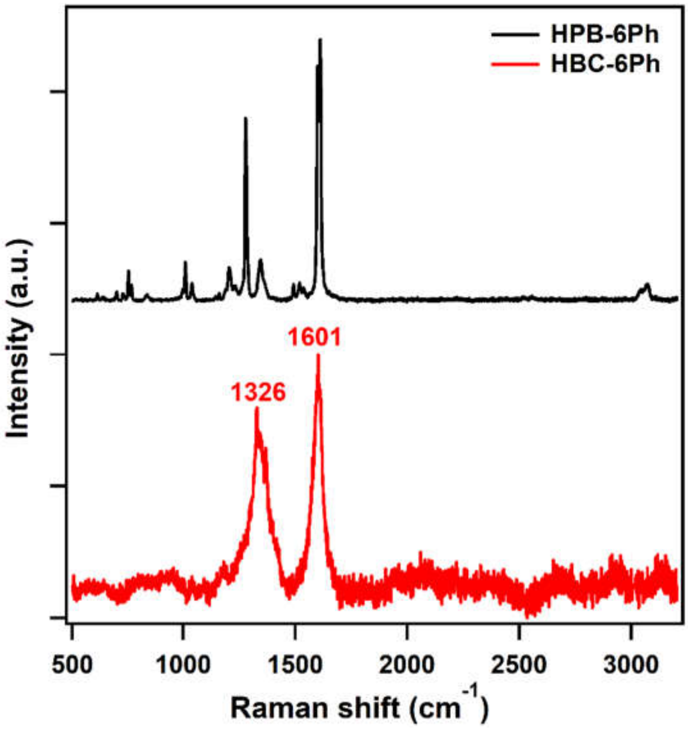

Figure S5. Raman spectra of **HPB-6Ph** and **HBC-6Ph**.

The D band stand for the disorder degree of carbon crystal structure like graphene. G band stems from in plane vibrations of carbon network. The existence of D band at 1326 cm^{-1} and G band at 1601 cm^{-1} of **HBC-6Ph** indicates that the electrodeposited films produce the graphene-like π-conjugated structures.

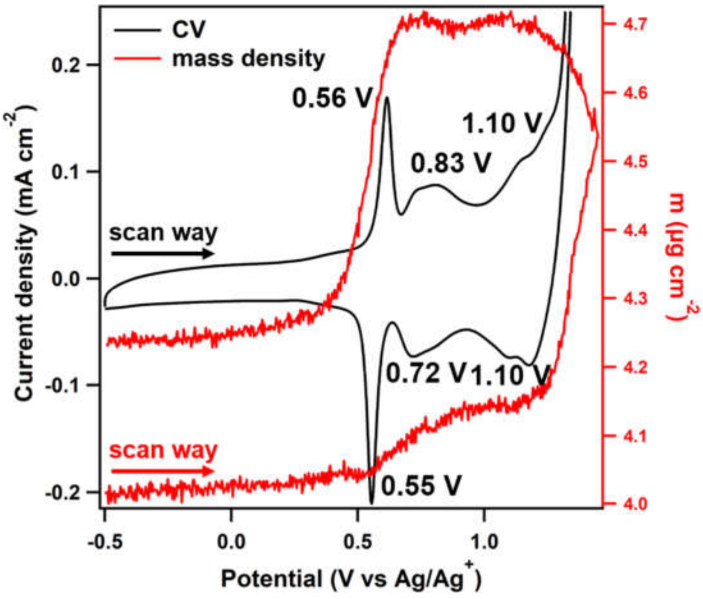

Figure S6. CV and EQCM profiles of a solution of **HPB-6Ph** (10th cycle).

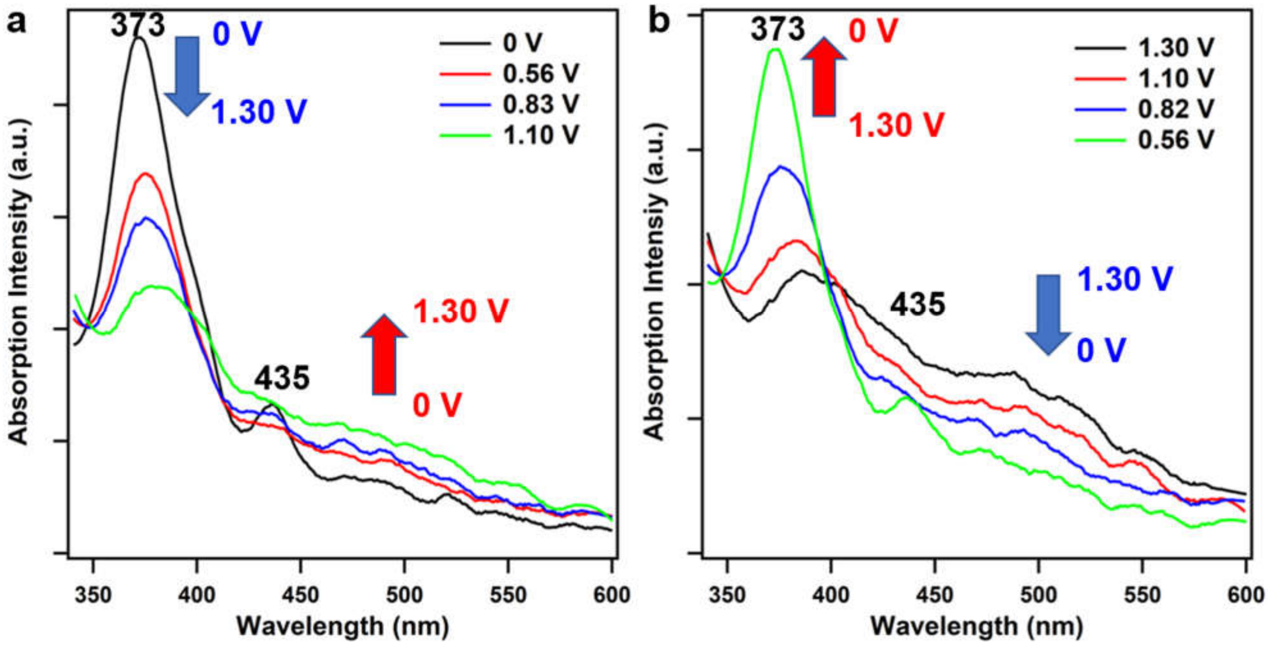

Figure S7. *In-situ* UV-vis absorption spectra of **HBC-6Ph** films. (a) Positive CV scan. (b) Negative CV scan.

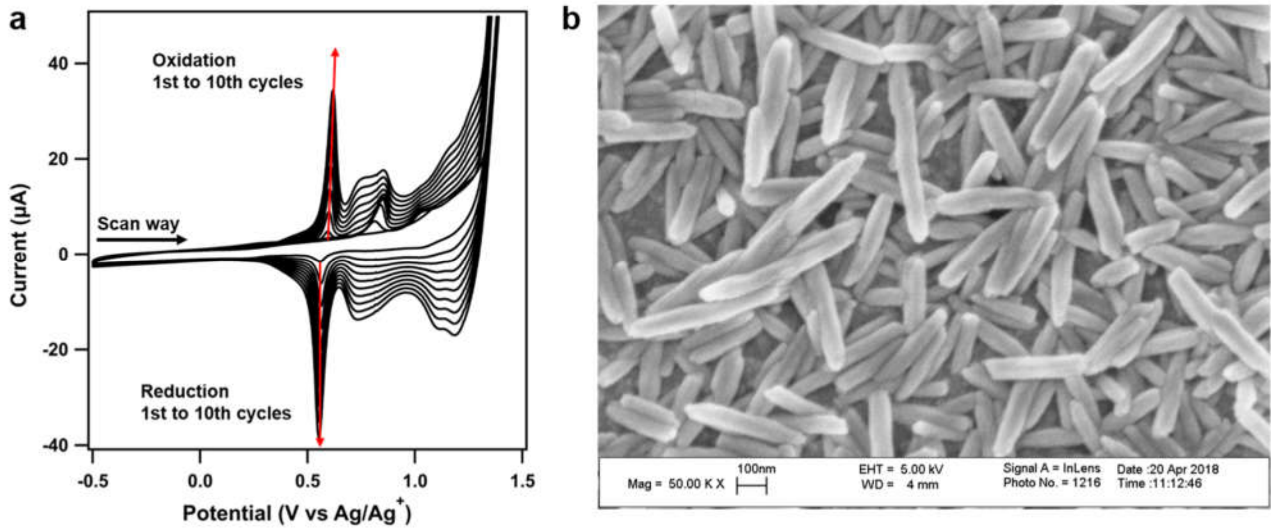

Figure S8. (a) CV profiles (1st to 10th cycles) of a solution of **HPB-6Ph** using Bu_4NPF_6 as supporting electrolyte with scan rate of 0.05 V s^{-1}. (b) SEM image of **HBC-6Ph** films using Bu_4NPF_6 as supporting electrolyte with scan rate of 0.05 V s^{-1}.

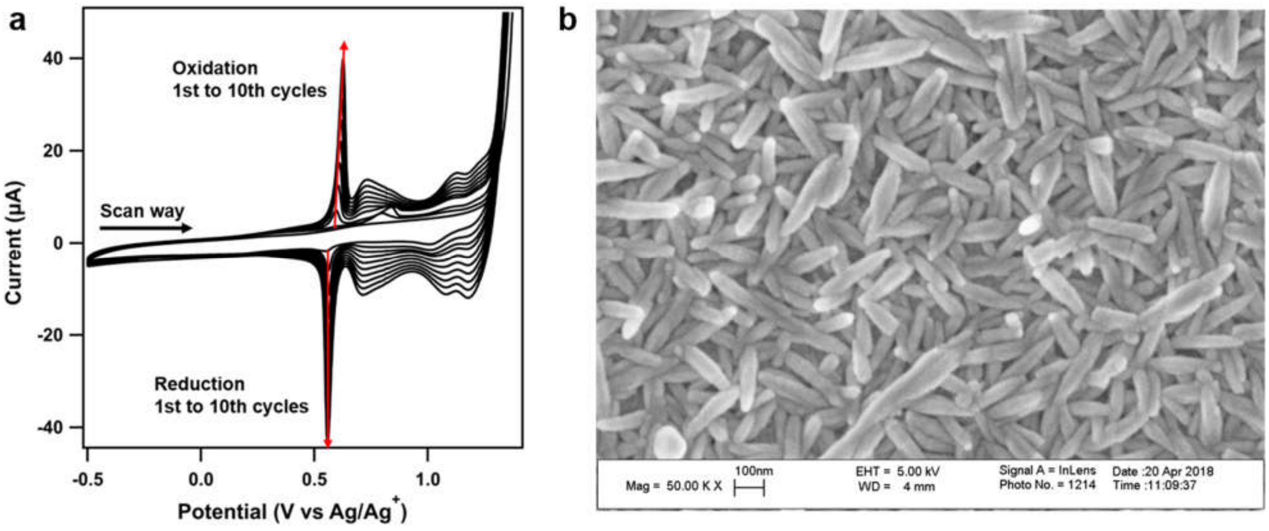

Figure S9. (a) CV profiles (1st to 10th cycles) of a solution of **HPB-6Ph** using Bu_4NPF_6 as supporting electrolyte with scan rate of 0.025 V s^{-1}. (b) SEM image of **HBC-6Ph** films using Bu_4NPF_6 as supporting electrolyte with scan rate of 0.025 V s^{-1}.

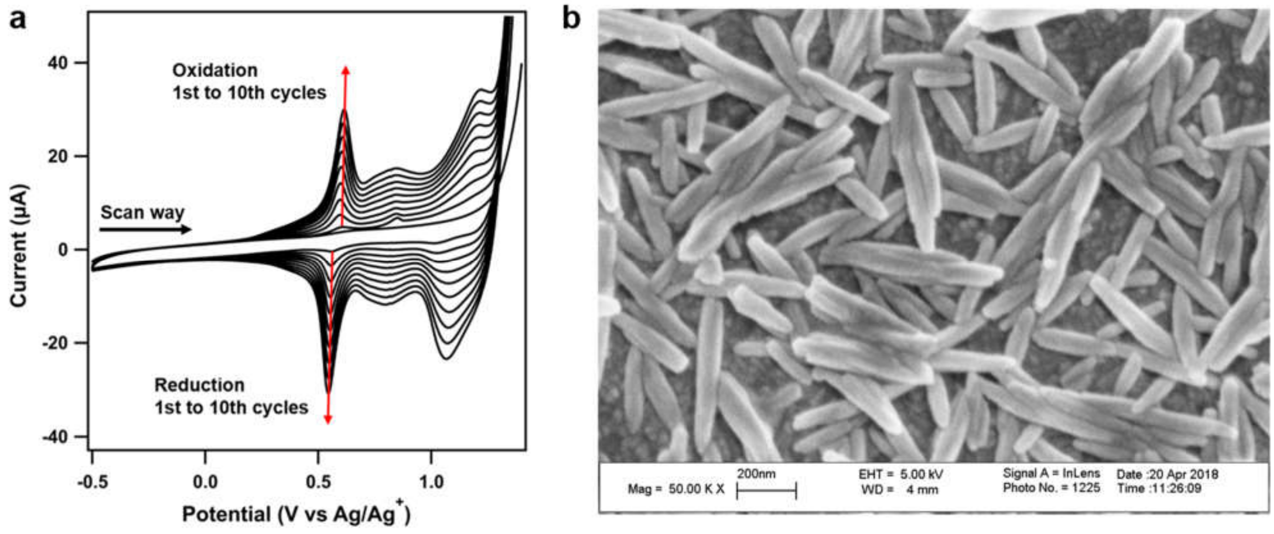

Figure S10. (a) CV profiles (1st to 10th cycles) of a solution of **HPB-6Ph** using Bu_4NPF_6 as supporting electrolyte with scan rate of 0.1 V s^{-1}. (b) SEM image of **HBC-6Ph** films using Bu_4NPF_6 as supporting electrolyte with scan rate of 0.1 V s^{-1}.

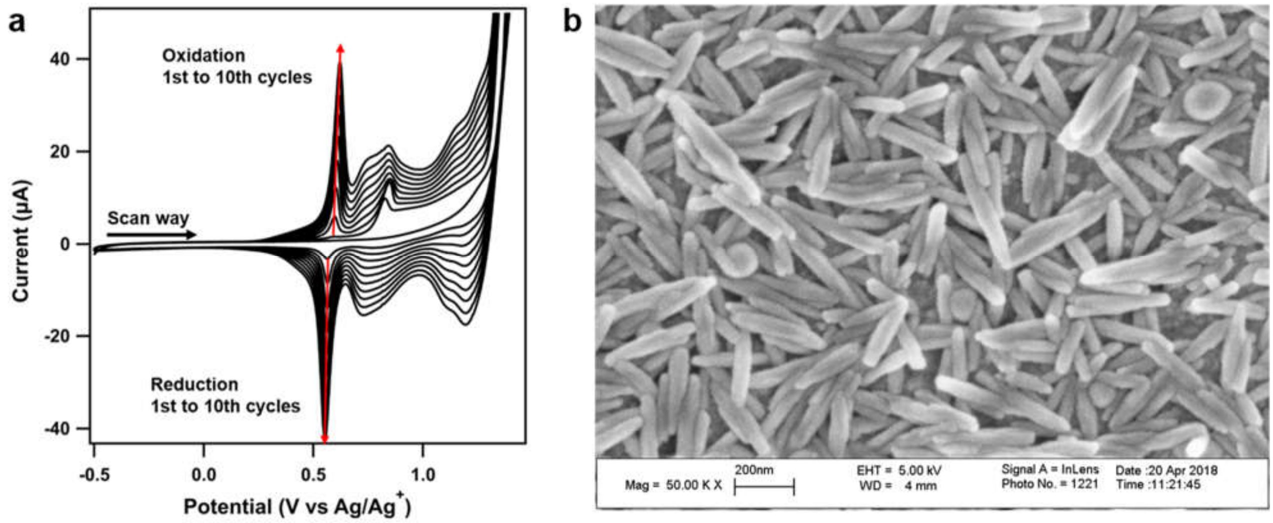

Figure S11. (a) CV profiles (1st to 10th cycles) of a solution of **HPB-6Ph** using Bu_4NAsF_6 as supporting electrolyte with scan rate of 0.05 V s^{-1}. (b) SEM image of **HBC-6Ph** films using Bu_4NAsF_6 as supporting electrolyte with scan rate of 0.05 V s^{-1}.

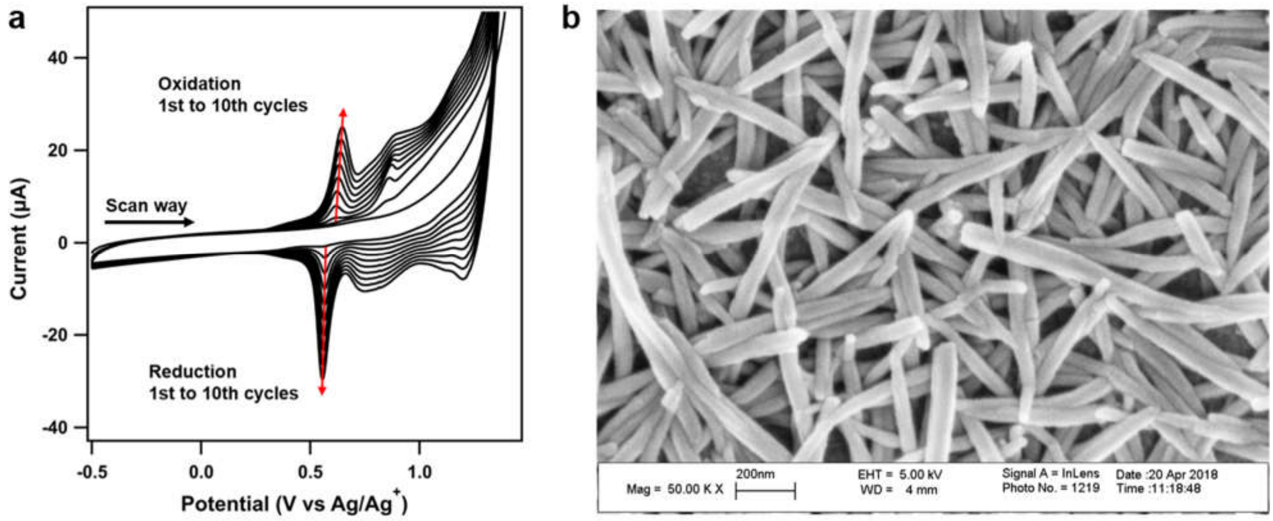

Figure S12. (a) CV profiles (1st to 10th cycles) of a solution of **HPB-6Ph** using Bu_4NBF_4 as supporting electrolyte with scan rate of 0.05 V s^{-1}. (b) SEM image of **HBC-6Ph** films using Bu_4NBF_4 as supporting electrolyte with scan rate of 0.05 V s^{-1}.

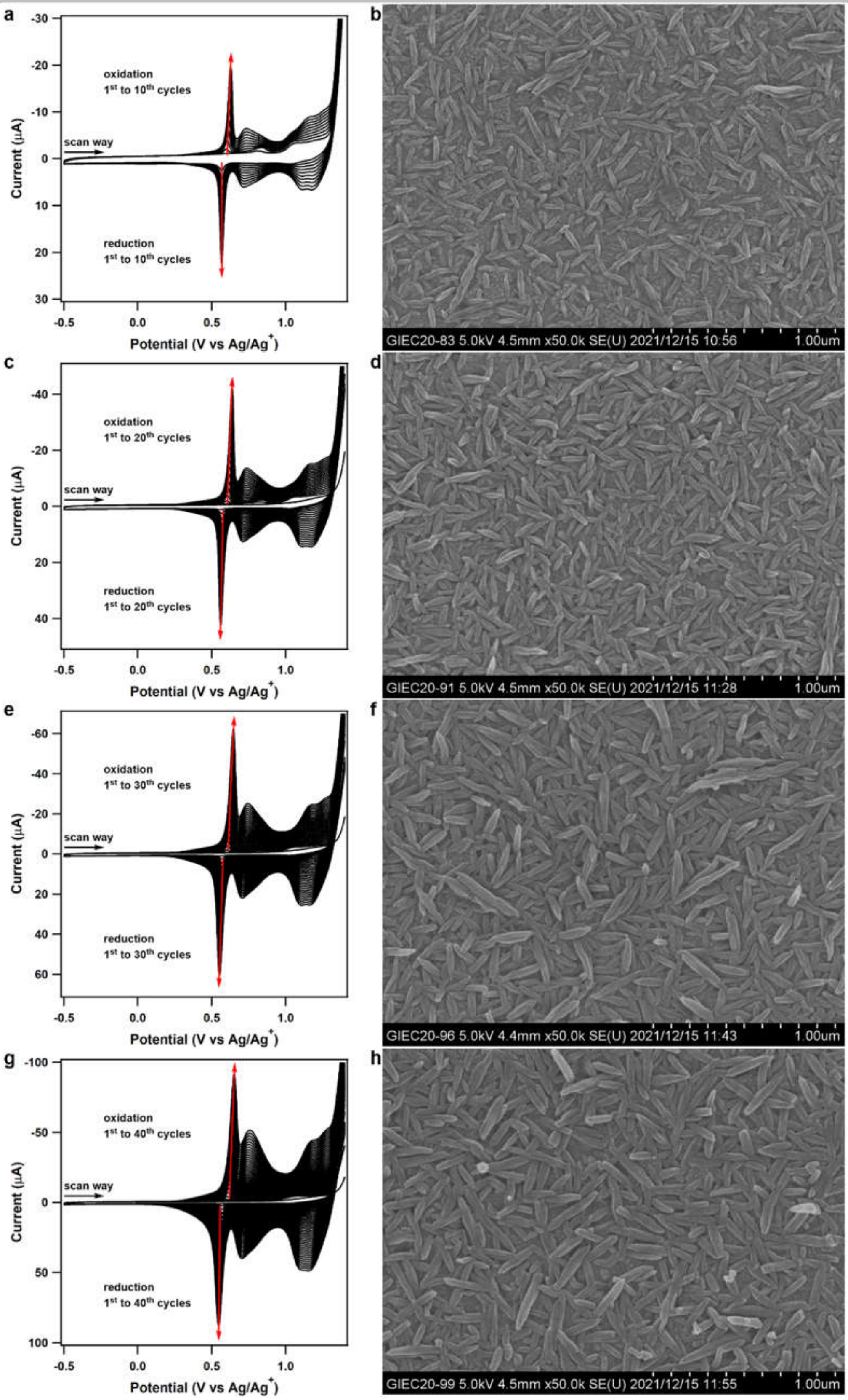

Figure S13. CV profiles of a solution of **HPB-6Ph** using Bu_4NPF_6 as supporting electrolyte with scan numbers of (a) 10, (c) 20, (e) 30, and (g) 40 cycles. SEM image of **HBC-6Ph** films using Bu_4NPF_6 as supporting electrolyte with scan numbers of (b) 10, (d) 20, (f) 30, and (h) 40 cycles.

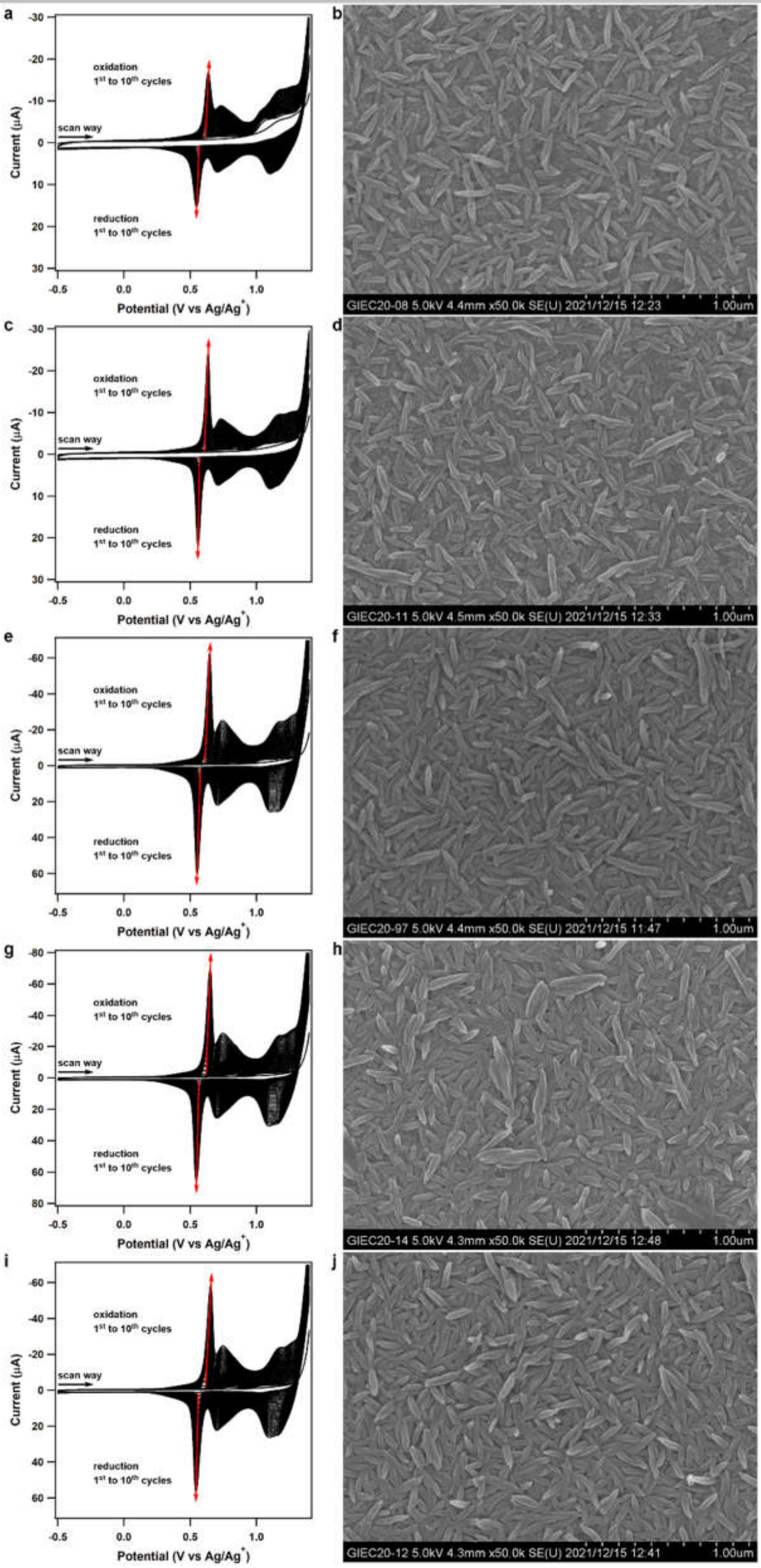

Figure S14. CV profiles (1st to 10th cycles) of a solution of **HPB-6Ph** using Bu_4NPF_6 as supporting electrolyte with the monomer concentrations of (a) 0.025, (c) 0.05, (e) 0.1, (g) 0.15, and (i) 0.2 mg mL^{-1}. SEM image of **HBC-6Ph** films using Bu_4NPF_6 as supporting electrolyte with the monomer concentrations of (b) 0.025, (d) 0.05, (f) 0.1, (h) 0.15, and (j) 0.2 mg mL^{-1}.

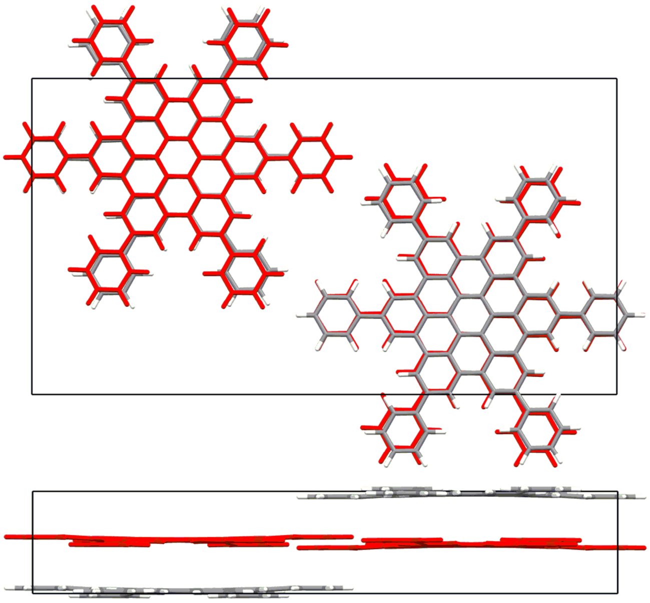

Figure S15. Top and side views of the **HBC-6Ph** in the complete staggered stacking mode.

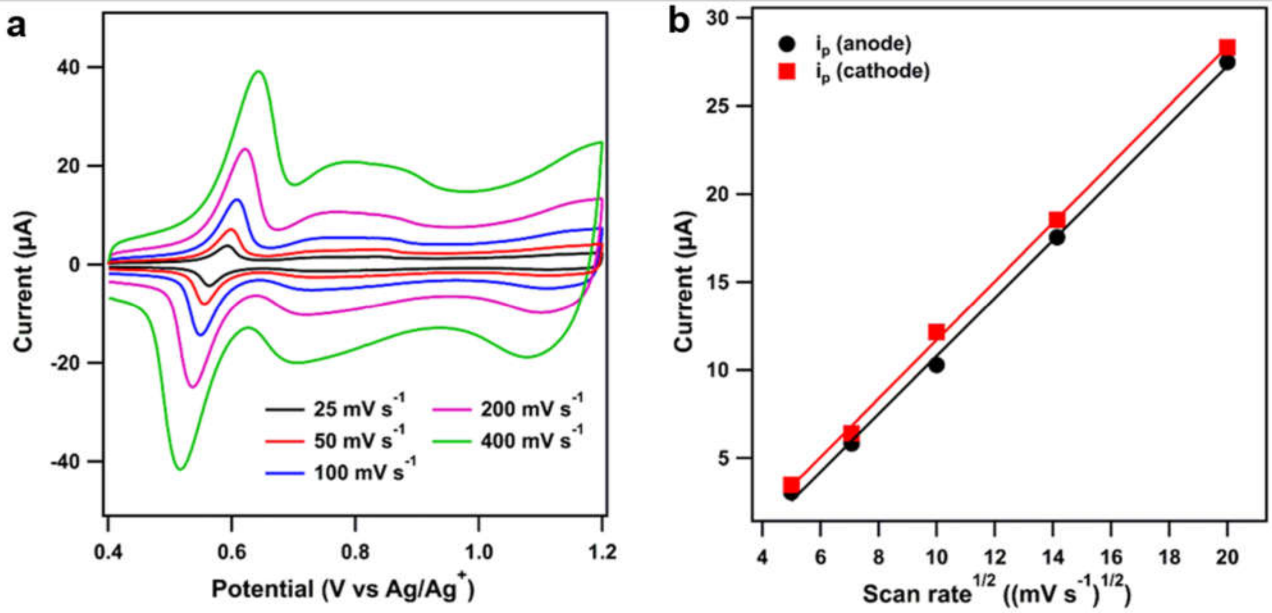

Figure S16. The electrochemical reaction rate-determining process of **HBC-6Ph** films (a) CV profiles of the 1st cycle of **HBC-6Ph** films in dichloromethane in the presence of the Bu_4NPF_6 electrolyte at 25 °C with a scan rate ranging from 0.01 V s^{-1} to 0.2 V s^{-1}. (b) Plot of the peak current versus the scan rate$^{1/2}$ of **HBC-6Ph** films.

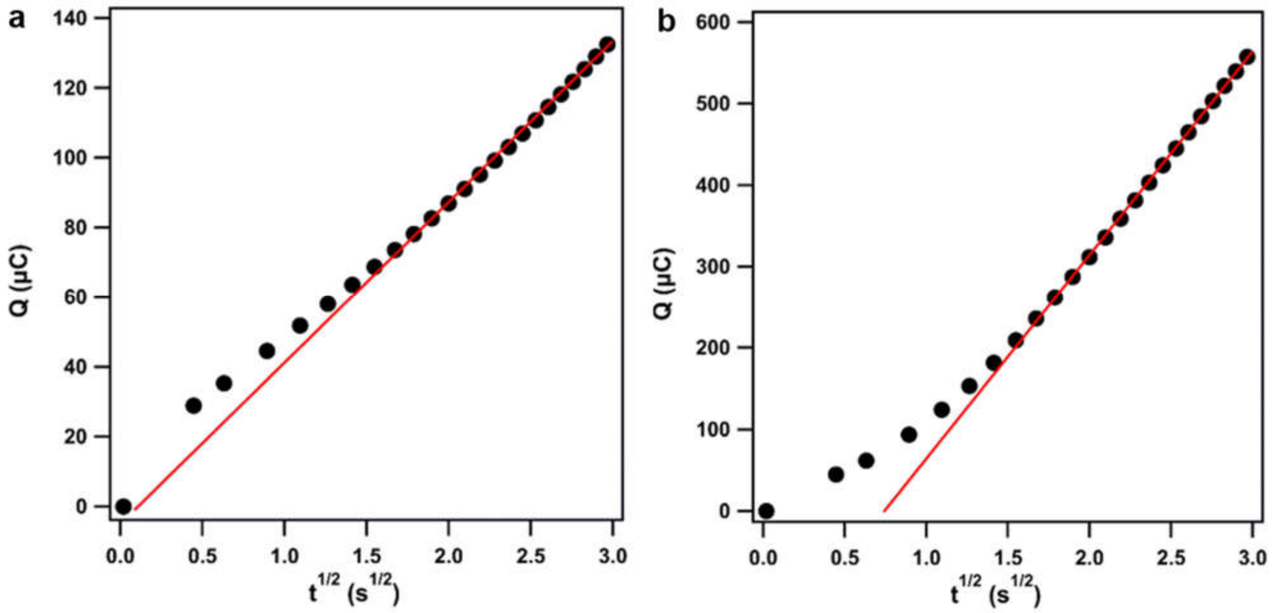

Figure S17. Plot of the electric quantity versus the time$^{1/2}$ of **HPB-6Ph** and **HPB** with 10 s at potential of 1.45 V, red line represents Cottrell equation fitting. (a) **HPB-6Ph**. (b) **HPB**.

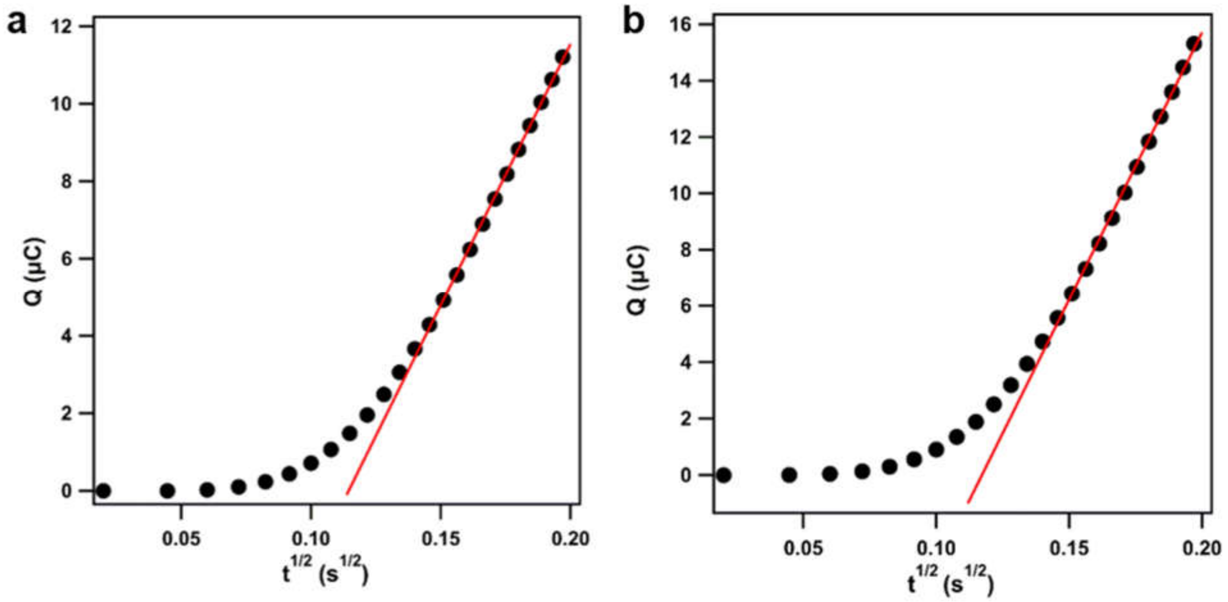

Figure S18. Plot of the electric quantity versus the time$^{1/2}$ of **HPB-6Ph** and **HPB** with 0.1 s at potential of 1.35 V, red line represents Cottrell equation fitting. (a) **HPB-6Ph**. (b) **HPB**.

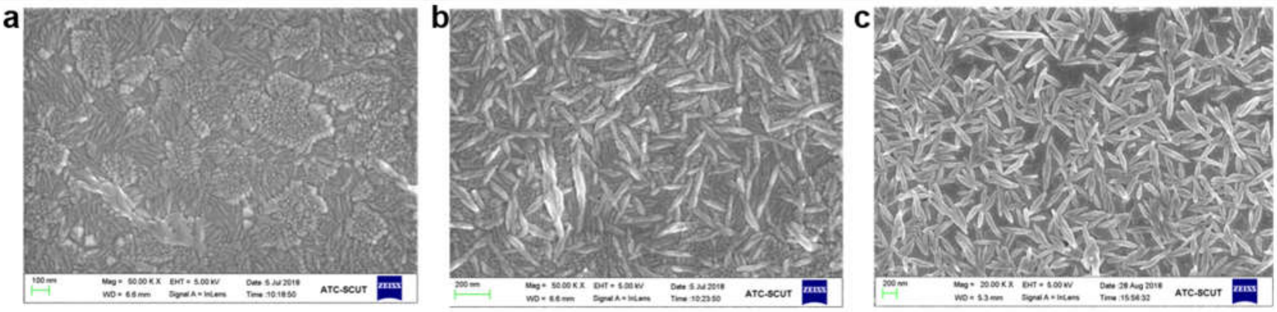

Figure S19. SEM images of **HBC-6Ph** films on ITO. (a) 1 cycle. (b) 2 cycles. (c) 10 cycles.

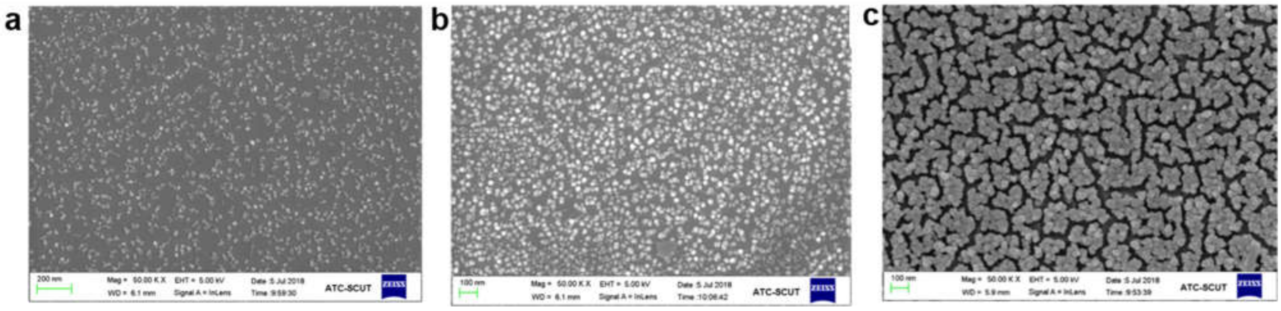

Figure S20. SEM images of **HBC-6Ph** films on MLG. (a) 1 cycle. (b) 2 cycles. (c) 10 cycles.

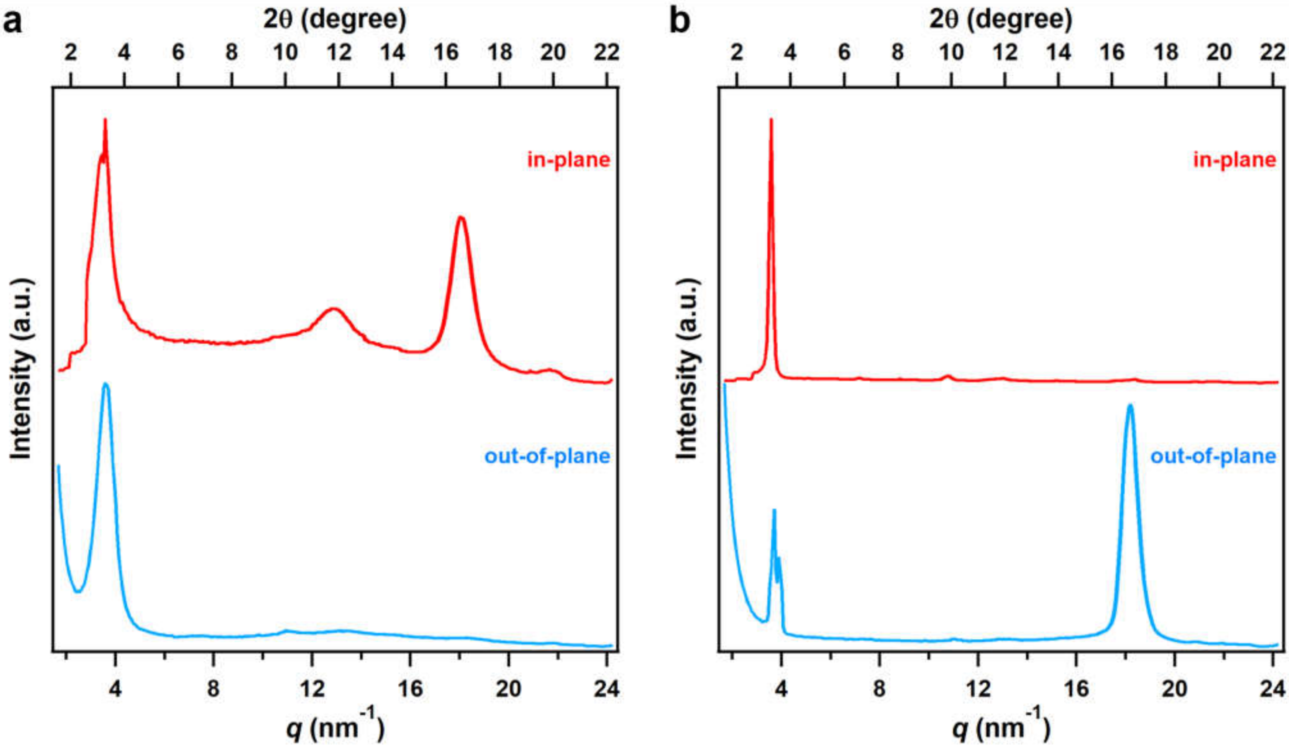

Figure S21. GIWAXS patterns extracted from the 2D images. (a) **HBC-6Ph** film on ITO electrode. (b) **HBC-6Ph** film on MLG electrode.

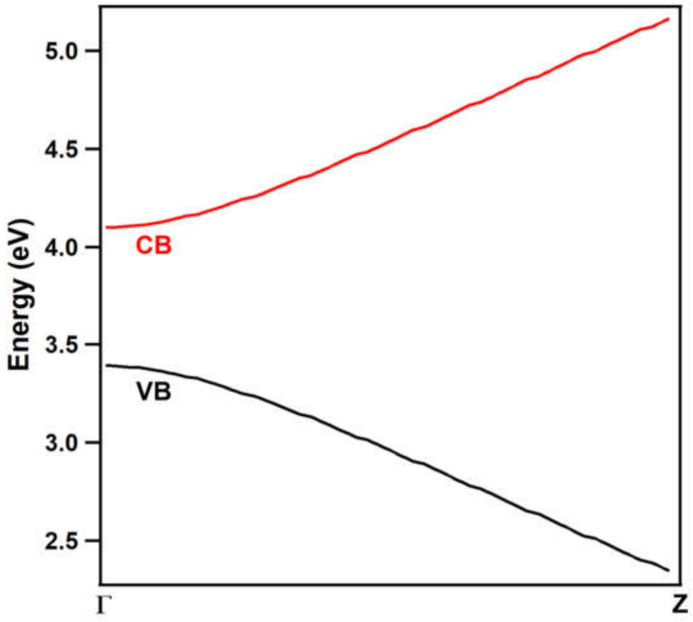

Figure S22. Calculated band dispersion of **HBC-6Ph** single crystal.

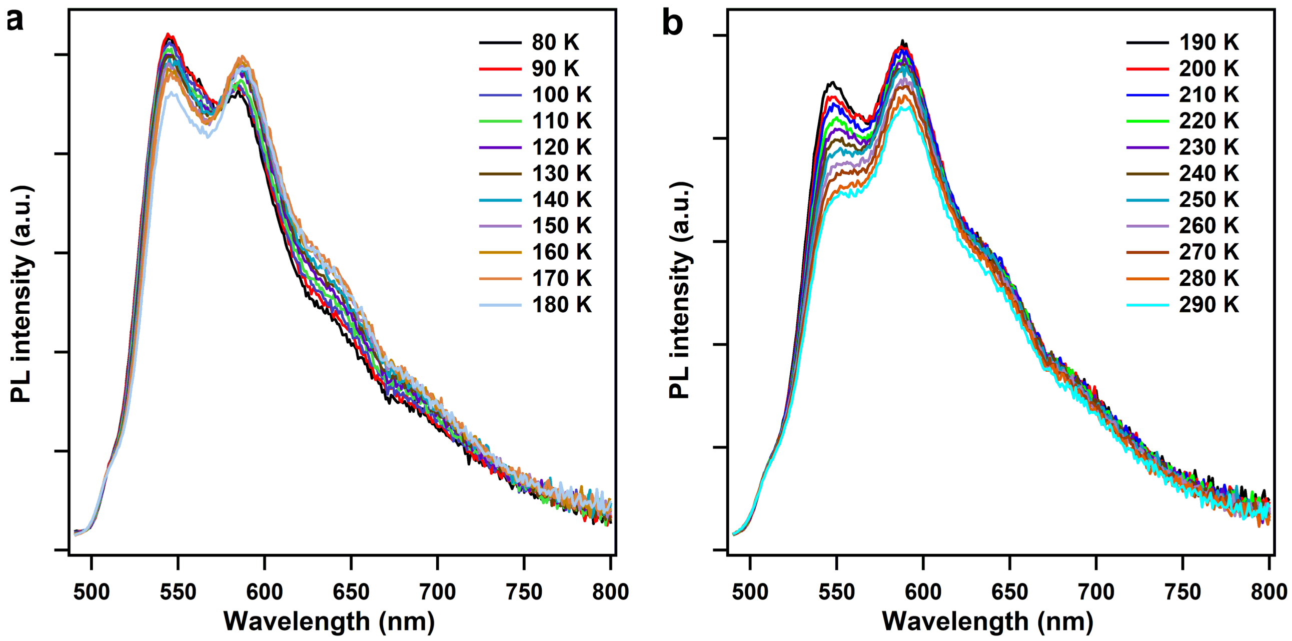

Figure S23. Temperature-dependent PL of **HBC-6Ph** films on ITO. (a) Temperature-dependent PL spectra in the range of 80 to 180 K. (b) Temperature-dependent PL spectra in the range of 190 to 290 K.

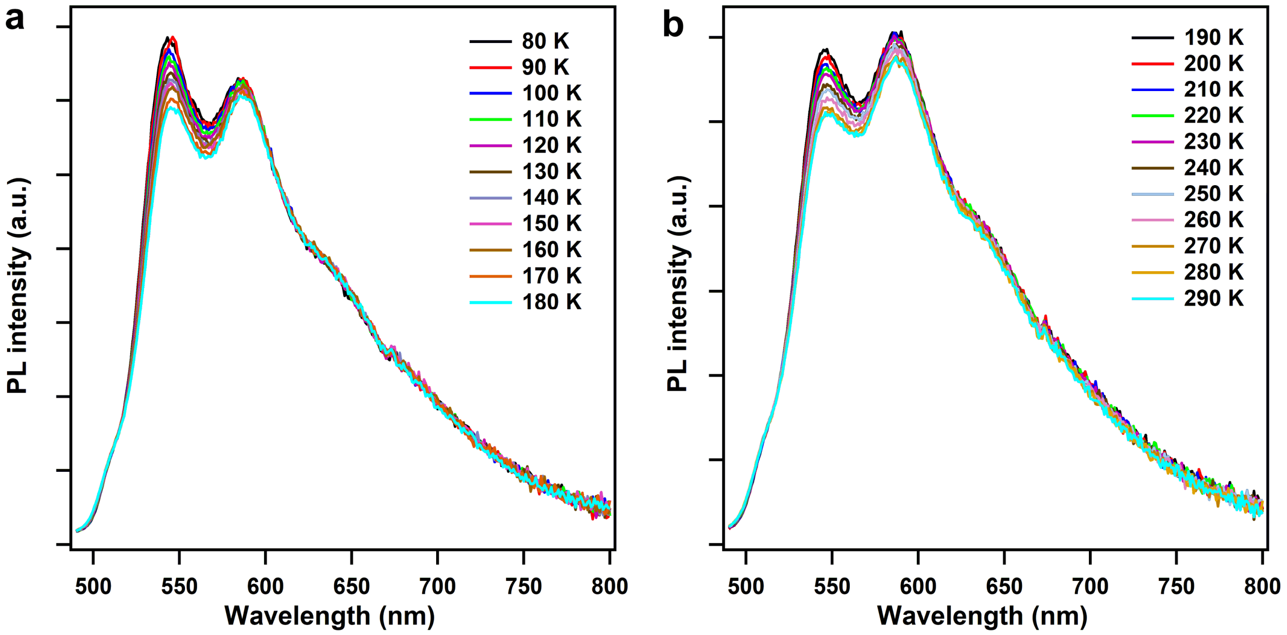

Figure S24. Temperature-dependent PL of **HBC-6Ph** films on MLG. (a) Temperature-dependent PL spectra in the range of 80 to 180 K. (b) Temperature-dependent PL spectra in the range of 190 to 290 K.

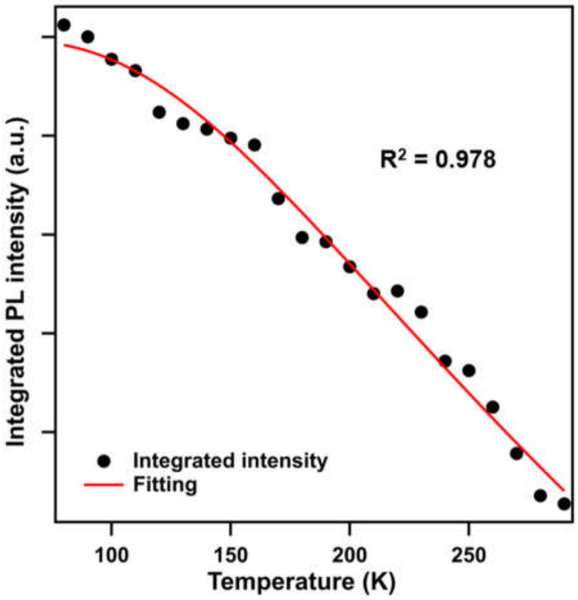

Figure S25. Temperature-dependent integrated PL intensity and fitting of **HBC-6Ph** films on MLG.

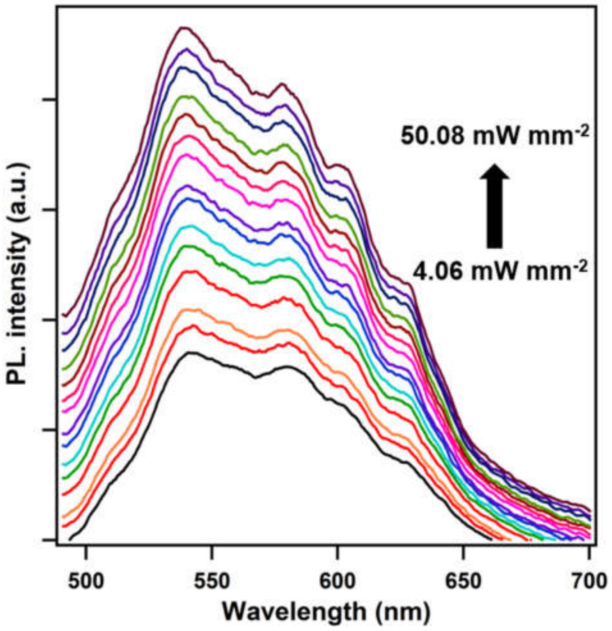

Figure S26. Power-dependent PL of **HBC-6Ph** films on ITO.

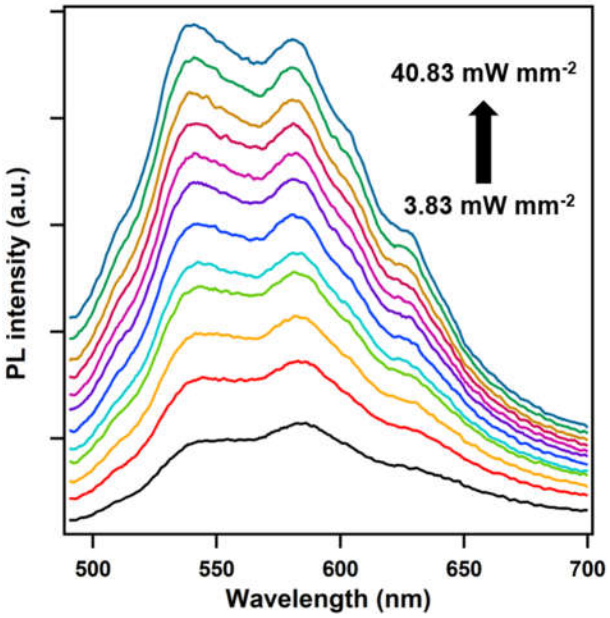

Figure S27. Power-dependent PL of **HBC-6Ph** films on MLG.

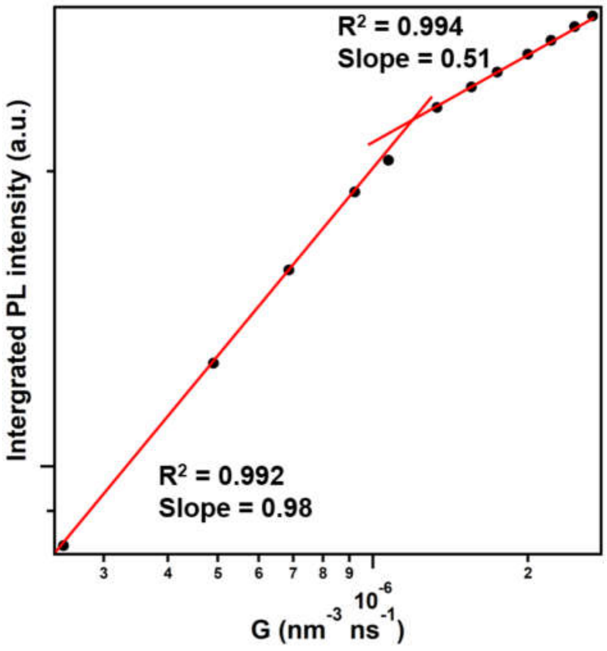

Figure S28. Plot of the integrated PL intensity versus exciton generation rate for **HBC-6Ph** films on MLG.

Tables

Table S1. Atomistic coordinates of completely eclipsed stack of **HBC-6Ph** optimized by using DFT method.
Space group: *PCCN;*
a = 37.6168 Å, *b* = 21.1325 Å, *c* = 7.0511 Å;
α = *β* = *γ* = 90˚.

Atom	x/a	y/b	z/c
C	-0.29197	0.51895	-1.01874
C	-0.3292	0.51912	-1.01895
C	-0.3459	0.5782	-1.01249
C	-0.38286	0.69873	-0.99099
C	-0.4018	0.7556	-0.9877
C	-0.38186	0.81158	-0.99148
C	-0.34145	0.93015	-1.00905
C	-0.32289	0.98749	-1.01831
C	-0.28574	0.984	-1.0211
C	-0.27174	0.57465	-1.01379
C	-0.32724	0.63531	-1.00559
C	-0.34581	0.69597	-0.99854
C	-0.34463	0.81203	-0.99802
C	-0.32443	0.87122	-1.00446
C	-0.23287	0.57319	-1.01411
C	-0.28944	0.63424	-1.00747
C	-0.32597	0.75329	-1.00021
C	-0.28654	0.86889	-1.00723
C	-0.26961	0.69244	-1.00467
C	-0.28777	0.75159	-1.00324
C	-0.26818	0.80914	-1.00457
H	-0.27825	0.47378	-1.02284
C	-0.35049	0.45979	-1.02716
H	-0.37468	0.57974	-1.01447
H	-0.39756	0.65464	-0.98724
C	-0.44134	0.75569	-0.98209
H	-0.39585	0.85649	-0.98905
H	-0.37029	0.93143	-1.00545
C	-0.3415	1.04961	-1.02321
H	-0.27069	1.02768	-1.02954
C	-0.03895	0.68766	-0.97925
C	-0.00191	0.68781	-0.97494
C	0.01686	0.74475	-0.97326
C	-0.00206	0.80152	-0.97601
C	-0.03907	0.80118	-0.98042
C	-0.17749	0.39293	-1.0193
C	-0.16025	0.33464	-1.01889
C	-0.12323	0.33147	-1.02247
C	-0.1038	0.38767	-1.0282
C	-0.12114	0.44596	-1.02893
C	-0.38797	0.4611	-1.02617
C	-0.40814	0.40598	-1.03773

C	-0.39152	0.3472	-1.05111
C	-0.35448	0.34453	-1.05115
C	-0.33437	0.39977	-1.03893
H	-0.05257	0.64219	-0.98045
H	0.01243	0.64294	-0.97281
H	0.04583	0.74485	-0.96992
H	0.01203	0.84666	-0.97504
H	-0.05266	0.84666	-0.98281
H	-0.20641	0.39294	-1.0131
H	-0.17605	0.29145	-1.01144
H	-0.10971	0.28595	-1.01711
H	-0.0748	0.38619	-1.03019
H	-0.10494	0.48864	-1.03338
H	-0.4021	0.50594	-1.01733
H	-0.43709	0.40897	-1.03837
H	-0.40733	0.30427	-1.0649
H	-0.34105	0.29908	-1.06482
H	-0.30552	0.39542	-1.04159

SUPPORTING INFORMATION

Table S2. Atomistic coordinates of rotated eclipsed stack of **HBC-6Ph** optimized by using DFT method.

Space group: *PCCN;*
a = 37.7272 Å, *b* = 20.3949 Å, *c* = 6.9802 Å;
α = *β* = *γ* = 90˚.

Atom	*x/a*	*y/b*	*z/c*
C	-0.25556	0.49874	-0.92906
C	-0.29139	0.48136	-0.92978
C	-0.31673	0.53123	-0.92268
C	-0.37018	0.63316	-0.90663
C	-0.39642	0.68142	-0.90241
C	-0.38602	0.74718	-0.90217
C	-0.36493	0.88439	-0.91134
C	-0.35533	0.95051	-0.91575
C	-0.31930	0.96644	-0.91869
C	-0.24459	0.56430	-0.92165
C	-0.30725	0.59745	-0.91607
C	-0.33411	0.64869	-0.91122
C	-0.35024	0.76548	-0.90670
C	-0.33965	0.83421	-0.91049
C	-0.20711	0.58174	-0.91768
C	-0.27066	0.61493	-0.91663
C	-0.32361	0.71579	-0.91037
C	-0.30292	0.85080	-0.91364
C	-0.26029	0.68284	-0.91370
C	-0.28660	0.73298	-0.91250
C	-0.27631	0.80014	-0.91318
H	-0.23607	0.45947	-0.93215
C	-0.30269	0.41207	-0.94129
H	-0.34448	0.51707	-0.92798
H	-0.37864	0.58225	-0.90221
C	-0.43421	0.66241	-0.89943
H	-0.40672	0.78428	-0.90190
H	-0.39302	0.87262	-0.90615
C	-0.38268	1.00243	-0.91938
H	-0.31218	1.01805	-0.92517
C	-0.04041	0.79972	-0.80118
C	-0.00485	0.81871	-0.79807
C	0.00614	0.87565	-0.89308
C	-0.01882	0.91345	-0.99174
C	-0.05438	0.89471	-0.99484
C	-0.12341	0.43641	-0.83140
C	-0.09788	0.38692	-0.83938
C	-0.06580	0.39761	-0.93511
C	-0.05905	0.45862	-1.01907
C	-0.08459	0.50807	-1.01191
C	-0.33392	0.39093	-0.84940
C	-0.34539	0.32610	-0.86604
C	-0.32591	0.28102	-0.97443

C	-0.29471	0.30127	-1.06529
C	-0.28312	0.36607	-1.04850
H	-0.04872	0.75591	-0.72331
H	0.01423	0.78894	-0.71889
H	0.03379	0.89094	-0.88822
H	-0.01054	0.95759	-1.06777
H	-0.07350	0.92409	-1.07481
H	-0.14805	0.42798	-0.75323
H	-0.10295	0.34027	-0.76764
H	-0.04574	0.35931	-0.94125
H	-0.03380	0.46759	-1.09090
H	-0.07942	0.55489	-1.08233
H	-0.34908	0.42547	-0.76239
H	-0.36958	0.31080	-0.79283
H	-0.33486	0.23042	-0.98824
H	-0.27971	0.26655	-1.15211
H	-0.25914	0.38185	-1.12308

Table S3. Electrochemical kinetic parameters of **HPB** and **HPB-6Ph**.

	**n**	**D_0 (×10^{-6} cm^2 s^{-1})**	**K_f (×10^{-2} cm s^{-1})**
**HPB**	12	1.53	2.2
**HPB-6Ph**	12	0.217	2.6

SUPPORTING INFORMATION

Table S4. Mobility of different organic semiconductor materials.

Material	State	Measurement method	Mobility ($cm^2\ V^{-1}\ s^{-1}$)	Ref.
C_{12}-BTBT 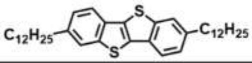	Film	FP-TRMC	170 (hole)	[S18]
Ph-BTBT-C_{10} 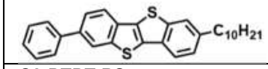	Film	FET	48.1 (hole)	[S19]
C8-BTBT:PS 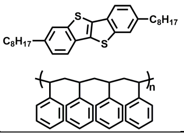	Film	FET	43 (hole)	[S20]
Rubrene 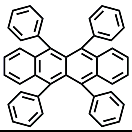	Crystal	FET	40 (hole)	[S21]
DPA 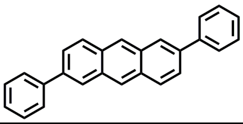	Crystal	FET	34 (hole)	[S22]
P3HT:PCBM 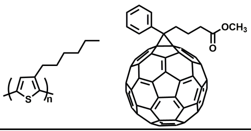	Film	THz	32	[S23]
**HBC-6Ph** 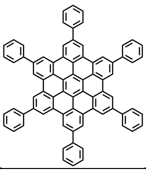	**Film on ITO**	**THz**	**30.9**	**This work**
TiOPc 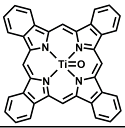	Crystal	FET	26.8 (hole)	[S24]
F_2TCNQ 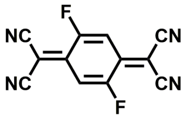	Crystal	FET	25 (hole)	[S25]
CDTBTZ 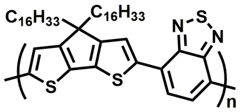	Film	FET	22.2 (hole)	[S26]
Pentacene 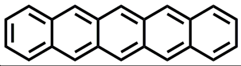	Film	THz	21 (hole)	[S27]
PDFDSe 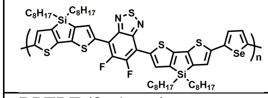	Film	FET	20.3 (hole)	[S28]
DBTDT (β-phase) 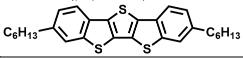	Crystal	FET	18.9 (hole)	[S29]
BTBTTBT	Crystal	FET	17.9 (hole)	[S30]

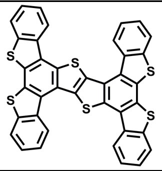				
F_4-BDOPV 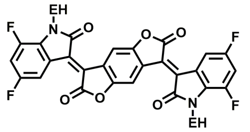	Crystal	FET	12.6 (hole)	[S31]
dNaAnt 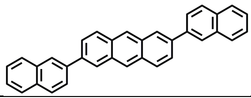	Crystal	FET	12.3 (hole)	[S32]
PDPP(SE)-ε-C_8C_{15} 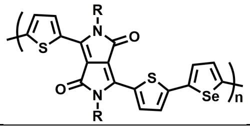	Film	FET	12.25 (hole)	[S33]
C_{10}-DNTT 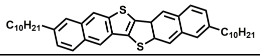	Film	Hall	11 (hole)	[S34]
PDBD-Se 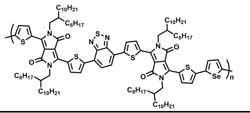	Film	FET	6.7 (hole)<br>4.3 (electron)	[S35]
PDPP2TzBDT 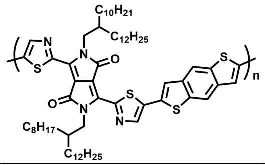	Crystal	FET	5.47 (hole)<br>5.33 (electron)	[S36]
P6F 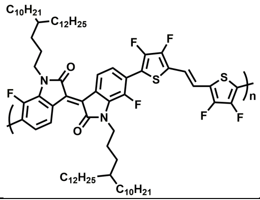	Film	FET	3.94 (hole)<br>3.5 (electron)	[S37]
INDTBT 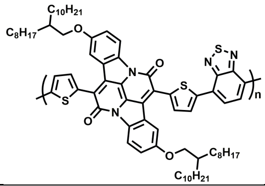	Film	FET	0.52 (hole)<br>3.1 (electron)	[S38]
4Cl-TAP 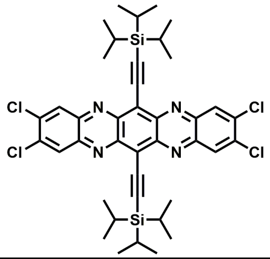	Film	FET	27.8 (electron)	[S39]
F_4BDOPV-2T	Film	FET	14.9 (electron)	[S40]

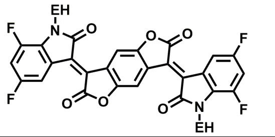				
Cl2-NDI 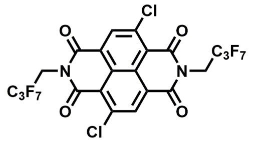	Crystal	FET	8.6 (electron)	[S41]
P4 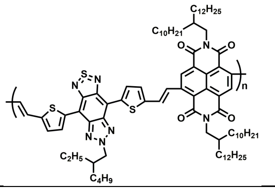	Film	FET	7.37 (electron)	[S42]
5,7-ICZ 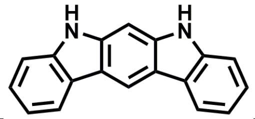	Crystal	FET	3.6 (electron)	[S43]
AzaBDOPV-2T 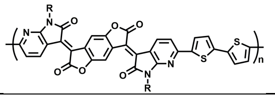	Film	FET	3.22 (electron)	[S44]
DPPPhF4 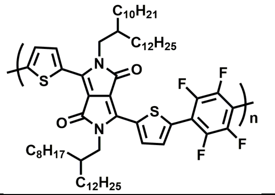	Film	FET	2.36 (electron)	[S45]

Table S5. Exciton binding energy of different organic semiconductor materials.

**Material**	**State**	**E_b (meV)**	**Ref.**
PAE-D 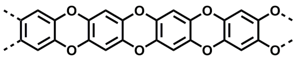	Powder	42	[S46]
a-PA 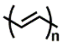	Crystal	50	[S47]
1,6-DTEP 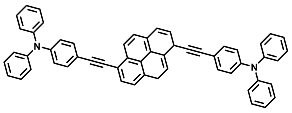	Crystal	59.22	[S48]
FSO-FS 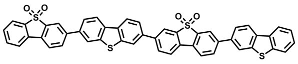	Powder	88	[S49]
DPYA-IFB 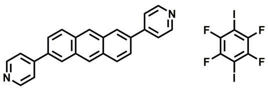	Crystal	95.9	[S50]
**HBC-6Ph** 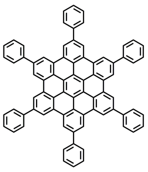	**Film on ITO**	**110**	**This work**
PTCDA	Film	150	[S51]

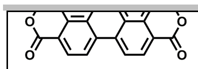			
PBnDT-DPNT P2F 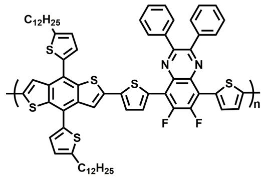	Film	180	[S52]
PPV 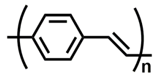	Crystal	200	[S53]
MDMO-PPV:PCBM 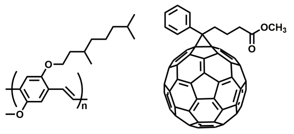	Film	203	[S54]
PffBT4T-2OD 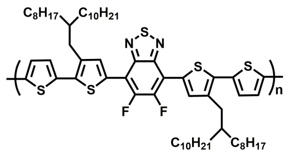	Film	300	[S55]
PFO 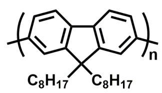	Film	300	[S56]
PBDTTT-EFT 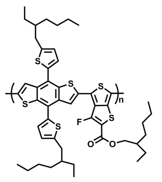	Film	360	[S55]

MEH-PPV 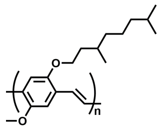	Film	360	[S56]
α-6T 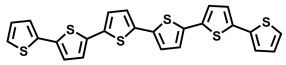	Film	400	[S56]
PPPV 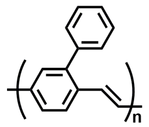	Film	400	[S58]
PDES 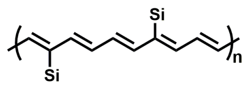	Film	500	[S59]
PDA 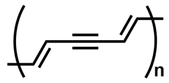	Film	500	[S60]
PDI-Y 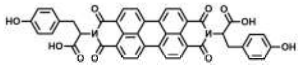	Film	510	[S61]
BTTIC-0M 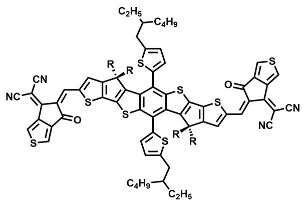	Crystal	540	[S62]
PT 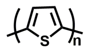	Film	600	[S59]

PTB7 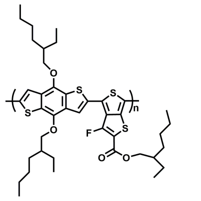	Film	710	[S63]
Biphenyl 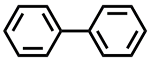	Crystal	720	[S64]
P3HT 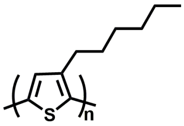	Film	730	[S63]
PCDTBT 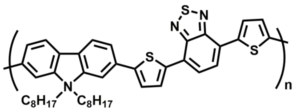	Film	740	[S63]
2,2'-bithiophene 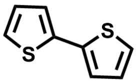	Crystal	780	[S64]
anthracene 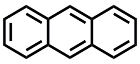	Crystal	1000	[S65]
α-NPD 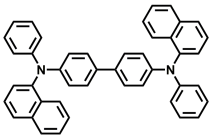	Film	1000	[S57]

Table S6. Exciton diffusion length of different organic semiconductor materials.

Material	State	Exciton diffusion length (nm)	Ref.
PDHF14-b-QPT22<br>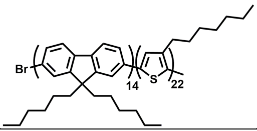	Crystal	210	[S66]
DIP<br>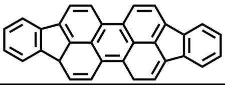	Crystal	100	[S67]
PBI<br>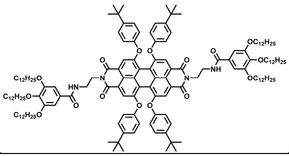	Crystal	96	[S68]
**HBC-6Ph**<br>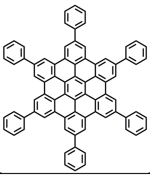	**Film on ITO**	**63**	**This work**
6T<br>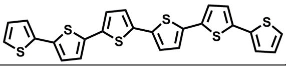	Crystal	60	[S69]
pFNI<br>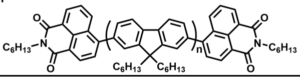	Film	34	[S70]
PffBT4T-2OD<br>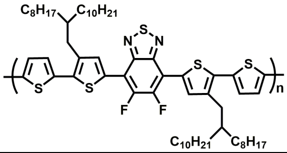	Film	24	[S71]
PTCDA<br>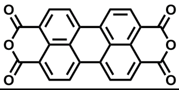	Crystal	21.5	[S72]
TPD<br>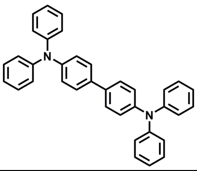	Film	17	[S73]
BP<br>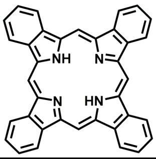	Crystal	15	[S74]
C6PT1C6-DPP<br>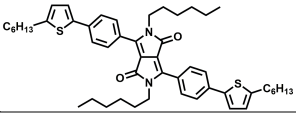	Crystal	12.9	[S75]
m-LPPP	Film	11	[S76]

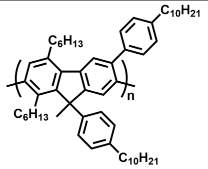			
ASSQ 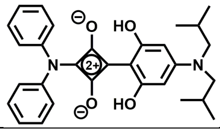	Film	11	[S77]
F12TBT 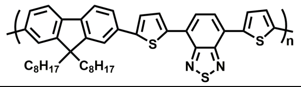	Film	11	[S78]
DPASQ 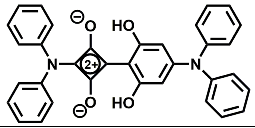	Film	10.7	[S77]
C–PCPDTBT 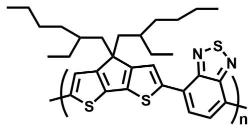	Film	10.5	[S79]
H_2TOPP 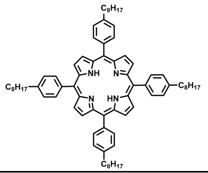	Film	9.6	[S80]
TBCM3PP 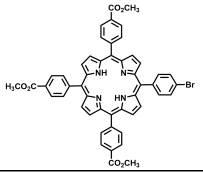	Film	9.4	[S81]
TFB 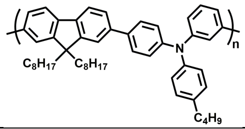	Film	9	[S82]
PEOPT 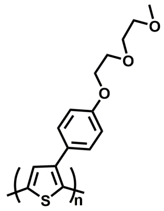	Film	8	[S83]
SubPc 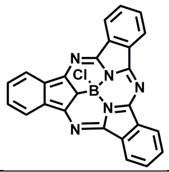	Film	7.7	[S84]
P3HT	Film	5.4	[S79]

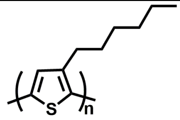			
NRS-PPV 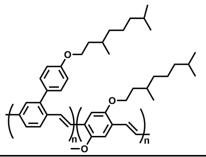	Film	5	[S85]
PCBM 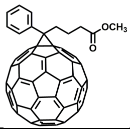	Film	5	[S86]
QQT(CN)4 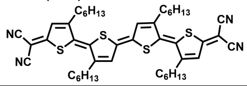	Crystal	5	[S87]
MDMO-PPV 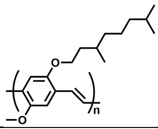	Film	4.5	[S88]
1- NPSQ 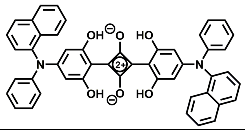	Film	2.9	[S77]
trans-PTCBI 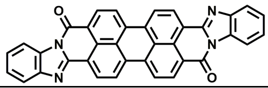	Film	1.3	[S89]

SUPPORTING INFORMATION

References

[1] L. Gao, W. Ren, H. Xu, L. Jin, Z. Wang, T. Ma, L.-P. Ma, Z. Zhang, Q. Fu, L.-M. Peng, X. Bao, H.-M. Cheng, *Nat. Commun.* **2012**, *3*, 699.
[2] O. A. González-Meza, E. R. Larios-Durán, A. Gutiérrez-Becerra, N. Casillas, J. I. Escalante, M. Bárcena-Soto, *J. Solid State Electrochem.* **2019**, *23*, 3123.
[3] S. J. Konopka, B. McDuffie, *Anal. Chem.* **1970**, *42*, 1741.
[4] B. R. Scharifker, J. Mostany, *J. Electroanal. Chem.* **1984**, *177*, 13.
[5] M. M. Islam, R. Kant, *Electrochim. Acta* **2011**, *56*, 4467.
[6] P. Hohenberg, W. Kohn, *Phys. Rev.* **1964**, *136*, B864.
[7] W. Kohn, L. J. Sham, *Phys. Rev.* **1965**, *140*, A1133.
[8] S. J. Clark, M. D. Segall, C. J. Pickard, P. J. Hasnip, M. J. Probert, K. Refson, M. C. Payne, *Z. Kristallogr.* **2005**, *220*, 567.
[9] J. P. Perdew, K. Burke, M. Ernzerhof, *Phys. Rev. Lett.* **1996**, *77*, 3865.
[10] S. Grimme, *J. Comput. Chem.* **2004**, *25*, 1463.
[11] S. Grimme, *J. Comput. Chem.* **2006**, *27*, 1787.
[12] B. Chmielak, M. Waldow, C. Matheisen, C. Ripperda, J. Bolten, T. Wahlbrink, M. Nagel, F. Merget, H. Kurz, *Opt. Express* **2011**, *19*, 17212.
[13] P. Giannozzi, S. Baroni, N. Bonini, M. Calandra, R. Car, C. Cavazzoni, D. Ceresoli, G. L. Chiarotti, M. Cococcioni, I. Dabo, A. D. Corso, S. Gironcoli, S. Fabris, G. Fratesi, R. Gebauer, U. Gerstmann, C. Gougoussis, A. Kokalj, M. Lazzeri, L. Martin-Samos, N. Marzari, F. Mauri, R. Mazzarello, S. Paolini, A. Pasquarello, L. Paulatto, Carlo. Sbraccia, S. Scandolo, G. Sclauzero, A. P. Seitsonen, A. Smogunov, P. Umari, R. M. Wentzcovitch, *J. Phys. Condens. Matter.* **2009**, *21*, 395502.
[14] D. R. Hamann, *Phys. Rev. B* **2013**, *88*, 085117.
[15] J. P. Perdew, A. Ruzsinszky, G. I. Csonka, O. A. Vydrov, G. E. Scuseria, L. A. Constantin, X. Zhou, K. Burke, *Phys. Rev. Lett.* **2008**, *100*, 136406.
[16] K. Lee, E. D. Murray, L. Kong, B. I. Lundqvist, D. C. Langreth, *Phys. Rev. B* **2010**, *82*, 081101.
[17] J. Zhou, X. Wen, N. Tang, X. Zhou, C. Wang, N. Zheng, L. Liu, Z. Xie, *J. Phys. Chem. Lett.* **2020**, *11*, 7908.
[18] Y. Tsutsui, G. Schweicher, B. Chattopadhyay, T. Sakurai, J.-B. Arlin, C. Ruzié, A. Aliev, A. Ciesielski, S. Colella, A. R. Kennedy, V. Lemaur, Y. Olivier, R. Hadji, L. Sanguinet, F. Castet, S. Osella, D. Dudenko, D. Beljonne, J. Cornil, P. Samorì, S. Seki, Y. H. Geerts, *Adv. Mater.* **2016**, *28*, 7106.
[19] T. Hamai, S. Arai, T. Hasegawa, *J. Mater. Res.* **2018**, *33*, 2350.
[20] Y. Yuan, G. Giri, A. L. Ayzner, A. P. Zoombelt, S. C. B. Mannsfeld, J. Chen, D. Nordlund, M. F. Toney, J. Huang, Z. Bao, *Nat. Commun.* **2014**, *5*, 3005.
[21] J. Takeya, M. Yamagishi, Y. Tominari, R. Hirahara, Y. Nakazawa, *Appl. Phys. Lett.* **2007**, *90*, 102120.
[22] J. Liu, H. Zhang, H. Dong, L. Meng, L. Jiang, L. Jiang, Y. Wang, J. Yu, Y. Sun, W. Hu, A. J. Heeger, *Nat. Commun.* **2016**, *6*, 10032.
[23] P. D. Cunningham, L. M. Hayden, *J. Phys. Chem. C* **2008**, *112*, 7928.
[24] Z. Zhang, L. Jiang, C. Cheng, Y. Zhen, G. Zhao, H. Geng, Y. Yi, L. Li, H. Dong, Z. Shuai, W. Hu, *Angew. Chem. Int. Ed.* **2016**, *55*, 5206.
[25] Y. Krupskaya, M. Gibertini, N. Marzari, A. F. Morpurgo, *Adv. Mater.* **2015**, *27*, 2453.
[26] C. Luo, A. K. K. Kyaw, L. A. Perez, S. Patel, M. Wang, B. Grimm, G. C. Bazan, E. J. Kramer, A. J. Heeger, *Nano Lett.* **2014**, *14*, 2764.
[27] S. G. Engelbrecht, M. Prinz, T. R. Arend, R. Kersting, *Appl. Phys. Lett.* **2014**, *105*, 012101.
[28] B. Nketia-Yawson, A.-R. Jung, H. D. Nguyen, K.-K. Lee, B. Kim, Y. Y. Noh, *ACS Appl. Mater. Interfaces* **2018**, *10*, 32492.
[29] P. He, Z. Tu, G. Zhao, Y. Zhen, H. Geng, Y. Yi, Z. Wang, H. Zhang, C. Xu, J. Liu, X. Lu, X. Fu, Q. Zhao, X. Zhang, D. Ji, L. Jiang, H. Dong, W. Hu, *Adv. Mater.* **2015**, *27*, 825.
[30] Y. Wang, S. Zou, J. Gao, H. Zhang, G. Lai, C. Yang, H. Xie, R. Fang, H. Li, W. Hu, *Chem. Commun.* **2015**, *51*, 11961.
[31] J.-H. Dou, Y.-Q. Zheng, Z.-F. Yao, Z.-A. Yu, T. Lei, X. Shen, X.-Y. Luo, J. Sun, S.-D. Zhang, Y.-F. Ding, G. Han, Y. Yi, J.-Y. Wang, J. Pei, *J. Am. Chem. Soc.* **2015**, *137*, 15947.
[32] J. Li, K. Zhou, J. Liu, Y. Zhen, L. Liu, J. Zhang, H. Dong, X. Zhang, L. Jiang, W. Hu, *J. Am. Chem. Soc.* **2017**, *139*, 17261.
[33] A.-R. Han, G. K. Dutta, J. Lee, H. R. Lee, S. M. Lee, H. Ahn, T. J. Shin, J. H. Oh, C. Yang, *Adv. Funct. Mater.* **2015**, *25*, 247.
[34] K. Nakayama, Y. Hirose, J. Soeda, M. Yoshizumi, T. Uemura, M. Uno, W. Li, M. J. Kang, M. Yamagishi, Y. Okada, E. Miyazaki, Y. Nakazawa, A. Nakao, K. Takimiya, J. Takeya, *Adv. Mater.* **2011**, *23*, 1626.
[35] Z. Ni, H. Wang, Q. Zhao, J. Zhang, Z. Wei, H. Dong, W. Hu, *Adv. Mater.* **2019**, *31*, 1806010.
[36] C. Xiao, G. Zhao, A. Zhang, W. Jiang, R. A. J. Janssen, W. Li, W. Hu, Z. Wang, *Adv. Mater.* **2015**, *27*, 4963.
[37] Y. Gao, Y. Deng, H. Tian, J. Zhang, D. Yan, Y. Geng, F. Wang, *Adv. Mater.* **2017**, *29*, 1606217.
[38] Kealan. J. Fallon, N. Wijeyasinghe, E. F. Manley, S. D. Dimitrov, S. A. Yousaf, R. S. Ashraf, W. Duffy, A. A. Y. Guilbert, D. M. E. Freeman, M. Al-Hashimi, J. Nelson, J. R. Durrant, L. X. Chen, I. McCulloch, T. J. Marks, T. M. Clarke, T. D. Anthopoulos, H. Bronstein, *Chem. Mater.* **2016**, *28*, 8366.
[39] M. Chu, J.-X. Fan, S. Yang, D. Liu, C. F. Ng, H. Dong, A.-M. Ren, Q. Miao, *Adv. Mater.* **2018**, *30*, 1803467.
[40] J.-H. Dou, Y.-Q. Zheng, Z.-F. Yao, T. Lei, X. Shen, X.-Y. Luo, Z.-A. Yu, S.-D. Zhang, G. Han, Z. Wang, Y. Yi, J.-Y. Wang, J. Pei, *Adv. Mater.* **2016**, *28*, 7213.
[41] T. He, M. Stolte, F. Würthner, *Adv. Mater.* **2013**, *25*, 6951.
[42] Y. Wang, T. Hasegawa, H. Matsumoto, T. Michinobu, *J. Am. Chem. Soc.* **2019**, *141*, 3566.
[43] H. Jiang, P. Hu, J. Ye, A. Chaturvedi, K. K. Zhang, Y. Li, Y. Long, D. Fichou, C. Kloc, W. Hu, *Angew. Chem. Int. Ed.* **2018**, *57*, 8875.
[44] Y.-Z. Dai, N. Ai, Y. Lu, Y.-Q. Zheng, J.-H. Dou, K. Shi, T. Lei, J.-Y. Wang, J. Pei, *Chem. Sci.* **2016**, *7*, 5753.
[45] J. H. Park, E. H. Jung, J. W. Jung, W. H. Jo, *Adv. Mater.* **2013**, *25*, 2583.
[46] Z.-A. Lan, M. Wu, Z. Fang, X. Chi, X. Chen, Y. Zhang, X. Wang, *Angew. Chem. Int. Ed.* **2021**, *60*, 16355.
[47] P. Puschnig, C. Ambrosch-Draxl, *Phys. Rev. Lett.* **2002**, *89*, 056405.
[48] J. Tao, D. Liu, Z. Qin, B. Shao, J. Jing, H. Li, H. Dong, B. Xu, W. Tian, *Adv. Mater.* **2020**, *32*, 1907791.
[49] Z.-A. Lan, G. Zhang, X. Chen, Y. Zhang, K. A. I. Zhang, X. Wang, *Angew. Chem. Int. Ed.* **2019**, *58*, 10236.
[50] G. Bolla, Q. Liao, S. Amirjalayer, Z. Tu, S. Lv, J. Liu, S. Zhang, Y. Zhen, Y. Yi, X. Liu, H. Fu, H. Fuchs, H. Dong, Z. Wang, W. Hu, *Angew. Chem. Int. Ed.* **2021**, *60*, 281.
[51] Z. Shen, P. E. Burrows, S. R. Forrest, M. Ziari, W. H. Steier, *Chem. Phys. Lett.* **1995**, *236*, 129.
[52] P. Yang, M. Yuan, D. F. Zeigler, S. E. Watkins, J. A. Lee, C. K. Luscombe, *J. Mater. Chem. C* **2014**, *2*, 3278.
[53] A. Ruini, M. J. Caldas, G. Bussi, E. Molinari, *Phys. Rev. Lett.* **2002**, *88*, 206403.
[54] J. Kern, S. Schwab, C. Deibel, V. Dyakonov, *Phys. Status Solidi RRL* **2011**, *5*, 364.
[55] H. Cha, C.-H. Tan, J. Wu, Y. Dong, W. Zhang, H. Chen, S. Rajaram, K. S. Narayan, I. McCulloch, J. R. Durrant, *Adv. Energy Mater.* **2018**, *8*, 1801537.

[56] S. F. Alvarado, P. F. Seidler, D. G. Lidzey, D. D. C. Bradley, *Phys. Rev. Lett.* **1998**, *81*, 1082.
[57] I. G. Hill, A. Kahn, Z. G. Soos, R. A. Pascal, Jr. *Chem. Phys. Lett.* **2000**, *327*, 181.
[58] R. Kersting, U. Lemmer, M. Deussen, H. J. Bakker, R. I. Mahrt, H. Kurz, V. I. Arkhipov, H. Bassler, E. O. Göbel, *Phys. Rev. Lett.* **1994**, *73*, 1440.
[59] M. Liess, S. Jeglinski, Z. V. Vardeny, M. Ozaki, K. Yoshino, Y. Ding, T. Barton, *Phys. Rev. B* **1997**, *56*, 15712.
[60] G. Weiser, *Phys. Rev. B* **1992**, *45*, 14076.
[61] Y.-O. Kim, B. J. Moon, A. Lee, J. I. Kim, S.-K. Lee, Y.-S. Lee, S. Bae, B. H. Hong, Y. C. Jung, *Adv. Optical Mater.* **2021**, 2101006.
[62] W. Gao, T. Liu, Z. Luo, L. Zhang, R. Ming, C. Zhong, W. Ma, H. Yan, C. Yang, *J. Mater. Chem. A* **2019**, *7*, 6809.
[63] H.-W. Li, Z. Guan, Y. Cheng, T. Lui, Q. Yang, C.-S. Lee, S. Chen, S.-W. Tsang, *Adv. Electron. Mater.* **2016**, *2*, 1600200.
[64] C. Ambrosch-Draxl, K. Hummer, S. Sagmeister, P. Puschnig, *Chem. Phys.* **2006**, *325*, 3.
[65] B. Schweitzer, H. Bässler, *Synth. Met.* **2000**, *109*, 1.
[66] X.-H. Jin, M. B. Price, J. R. Finnegan, C. E. Boott, J. M. Richter, A. Rao, S. M. Menke, R. H. Friend, G. R. Whittell, I. Manners, *Science* **2018**, *360*, 897.
[67] A. K. Topczak, T. Roller, B. Engels, W. Brutting, J. Pflaum, *Phys. Rev. B* **2014**, *89*, 201203.
[68] H. Marciniak, X. Q. Li, F. Würthner, S. Lochbrunner, *J. Phys. Chem. A* **2011**, *115*, 648.
[69] A. Mani, J. Schoonman, A. Goossens, *J. Phys. Chem. B* **2005**, *109*, 4829.
[70] Zaikowski, L. *et al.* Charge transfer fluorescence and 34 nm exciton diffusion length in polymers with electron acceptor end traps. *J. Phys. Chem. B* **119**, 7231−7241 (2015).
[71] L. Zaikowski, G. Mauro, M. Bird, B. Karten, S. Asaoka, Q. Wu, A. R. Cook, J. R. Miller, *Chem. Mater.* **2019**, *31*, 6548.
[72] R. R. Lunt, J. B. Benziger, S. R. Forrest, *Adv. Mater.* **2010**, *22*, 1233.
[73] C. L. Yang, Z. K. Tang, W. K. Ge, Z. L. Zhan, X. Y. Jian, *Appl. Phys. Lett.* **2003**, *83*, 1737.
[74] M. Guide, J. D. A. Lin, C. M. Proctor, J. Chen, C. García-Cervera, T.-Q. Nguyen, *J. Mater. Chem. A* **2014**, *2*, 7890.
[75] J. D. A. Lin, O. V. Mikhnenko, J. Chen, Z. Masri, A. Ruseckas, A. Mikhailovsky, R. P. Raab, J. Liu, P. W. M. Blom, M. A. Loi, C. J. García-Cervera, I. D. W. Samueld, T.-Q. Nguyen, *Mater. Horiz.* **2014**, *1*, 280.
[76] V. Gulbinasa,I. Minevičiūtė, D. Hertel, R. Wellander, A. Yartsev, V. Sundström, *J. Chem. Phys.* **2007**, *127*, 144907.
[77] K. J. Bergemann, S. R. Forrest, *Appl. Phys. Lett.* **2011**, *99*, 243303.
[78] C. Leow, T. Ohnishi, M. Matsumura, *J. Phys. Chem. C* **2013**, *118*, 71.
[79] O. V. Mikhnenko, H. Azimi, M. Scharber, M. Morana, P. W. M. Blom, M. A. Loi, *Energy Environ. Sci.* **2012**, *5*, 6960.
[80] J. E. Kroeze, R. B. M. Koehorst, T. J. Savenije, *Adv. Funct. Mater* **2004**, *14*, 992 (2004).
[81] A. L. Ortiz, G. C. Collier, D. M. Marin, J. A. Kassel, R. J. Ivins, N. G. Grubich, M. G. Walter, *J. Mater. Chem. C* **2015**, *3*, 1243.
[82] A. Bruno, L. X. Reynolds, C. Dyer-Smith,J. Nelson, S. A. Haque, *J. Phys. Chem. C* **2013**, *117*, 19832.
[83] L. A. A. Pettersson, L. S. Roman, O. Inganäs, *J. Appl. Phys.* **1999**, *86*, 487.
[84] W. A. Luhman, R. J. Holmes, *Adv. Funct. Mater.* **2011**, *21*, 764.
[85] D. E. Markov, E. Amsterdam, P. W. M. Blom, A. B. Sieval, J. C. Hummelen, *J. Phys. Chem. A* **2005**, *109*, 5266.
[86] S. Cook, A. Furube, R. Katoh, L. Han, *Chem. Phys. Lett.* **2009**, *478*, 33.
[87] H.-Y. Shin, J. H. Woo, M. J. Gwon, M. Barthelemy, M. Vomir, T. Muto, K. Takaishi, M. Uchiyama, D. Hashizume, T. Aoyama, D.-W. Kim, S. Yoon, J.-Y. Bigot, J. W. Wua, J. C. Ribierre, *Phys. Chem. Chem. Phys.* **2013**, *15*, 2867.
[88] O. V. Mikhnenko, F. Cordella, A. B. Sieval, J. C. Hummelen, P. W. M. Blom, M. A. Loi, *J. Phys. Chem. B* **2008**, *112*, 11601.
[88] S. B. Rim, R. F. Fink, J. C. Schöneboom, P. Erk, P. Peumans, *Appl. Phys. Lett.* **2007**, *91*, 173504.

Author Contributions

C.Z. conducted the electrochemical experiments and characterization. C.Z., and W.Z. performed the THz experiments and characterization. H.X. performed the structural simulation. S.O. calculated the effective mass. H.W. and B.M. directed the THz research. Z.Q. and K.M. conducted the mass measurement and gave suggestions on manuscript writing. K.O. measured synchrotron GIWAXS. W.M., W.R. and H.C. fabricated the monolayer graphene. C.G. and Y.M. conceived, supervised and supported the project. All authors contributed to the writing and editing of the manuscript. C.Z., W.Z. and H.X. contributed equally to this work.
